# Reprogramming the Tumor Immune Microenvironment with ICAM‐1‐Targeted Antibody‒Drug Conjugates and B7‐H3‐CD3 Bispecific Antibodies

**DOI:** 10.1002/advs.202415577

**Published:** 2025-02-25

**Authors:** Shoubing Zhou, Mengyu Hong, Dan Zhao, Wenyu Li, Xiaolong Yuan, Yinghong Wang, Hualong Li, Yang Yang, Tengchuan Jin, Jing Pan

**Affiliations:** ^1^ Department of Breast Oncology The First Affiliated Hospital of USTC Division of Life Sciences and Medicine University of Science and Technology of China Hefei Anhui 230031 China; ^2^ Department of Breast Oncology Anhui Provincial Cancer Hospital Hefei Anhui 230031 China; ^3^ Laboratory of Structural Immunology CAS Key Laboratory of Innate Immunity and Chronic Disease School of Basic Medical Sciences Division of Life Sciences and Medicine University of Science and Technology of China Hefei 230027 China; ^4^ Department of Tumor Radiotherapy People Hospital of Fengyang County Chuzhou Anhui 233100 China; ^5^ Department of Oncology The First Affiliated Hospital of Anhui Medical University Hefei 230022 China; ^6^ Department of Oncology Suzhou Hospital of Anhui Medical University Suzhou 234000 China; ^7^ Department of Obstetrics and Gynecology The First Affiliated Hospital of USTC Division of Life Sciences and Medicine Center for Advanced Interdisciplinary Science and Biomedicine of IHM University of Science and Technology of China Hefei Anhui 230001 China; ^8^ Institute of Health and Medicine, Hefei Comprehensive National Science Center Hefei Anhui China; ^9^ Biomedical Sciences and Health Laboratory of Anhui Province University of Science & Technology of China Hefei 230027 China; ^10^ Clinical Research Hospital of the Chinese Academy of Sciences (Hefei) University of Science and Technology of China Hefei 230001 China

**Keywords:** antibody‒drug conjugates, B7‐H3‐CD3, ICAM‐1, triple‐negative breast cancer, tumor immune microenvironment

## Abstract

Reprogramming the tumor immune microenvironment (TIM) plays an important role in promoting the reversal of immune “cold” tumors into “hot” inflammatory tumors. Improving drug targeting, blocking immune checkpoints, and promoting the activation of immune cells are crucial for reprogramming the TIM. Here, an intercellular adhesion molecule 1‐targeted antibody‒drug conjugate in combination with a B7‐H3‐CD3 bispecific antibody is selected for TIM reprogramming, which improved the efficacy of triple‐negative breast cancer immunotherapy. This combination therapy improves drug targeting, blocks immune checkpoint pathways, and activates effector T cells to release cytokines, leading to immunogenic cell death and the release of tumor‐associated antigens. This effect promotes the maturation of dendritic cells, infiltration and activation of cytotoxic CD8+ T cells, repolarization of M1‐type macrophages, and reduction of M2‐type macrophages, immune suppressor Tregs, and MDS cells, thereby reprogramming the TIM. In addition, this innovative strategy promotes the accumulation of immune cells at metastasis sites and significantly impedes the progression of lung metastatic lesions. Overall, this study provides novel insights for reprogramming the TIM using novel immunotherapeutic strategies that leverage the synergistic effects of antibody‐drug conjugates and bispecific antibodies.

## Graphical abstract

Reprogramming the tumor immune microenvironment is crucial for transforming immune “cold” tumors into “hot” inflammatory tumors. The investigation reveals that the combination of ICAM‐1‐Dxd with B7‐H3‐CD3 bispecific antibodies not only demonstrates significant inhibitory effects on tumor growth and metastasis but also plays a vital role in reprogramming the tumor immune microenvironment in triple‐negative breast cancer.

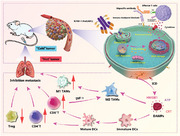

## 1 Background

Triple‐negative breast cancer (TNBC) is a particularly aggressive subtype of breast cancer characterized by resistance to standard treatment protocols and a poor prognosis. Patients with TNBC face the highest risk of recurrence and metastasis among all those with breast cancer.^[^ ^1^ ^]^ Although chemotherapy is the mainstay of treatment for TNBC, most patients experience recurrence and metastasis after the initial treatment.^[^ ^2^ ^]^ Recently, the therapeutic landscape for TNBC has been updated with the inclusion of olaparib^[^ ^3^ ^]^ and niraparib,^[^ ^4^ ^]^ which the National Comprehensive Cancer Network recommends for locally advanced or metastatic TNBC patients with genetic mutations. In addition, sacituzumab govitecan, an antibody‒drug conjugate that targets TROP2, is now recommended for patients who have undergone two or more prior treatment regimens.^[^ ^5^ ^]^ As a new antitumor therapeutic strategy, immunotherapy has achieved breakthrough progress in treating some solid tumors.^[^ ^6^, ^7^ ^]^ However, immunotherapies have shown suboptimal efficacy against advanced TNBC, possibly because of the tumor immunosuppressive microenvironment (TIM). Therefore, novel targets or therapeutic strategies that can enhance the efficacy of immunotherapy by reprogramming the TIM are urgently needed.

Reshaping the TIM is a critical challenge in enhancing the efficacy of cancer immunotherapy. Researchers previously reported successfully screening a high‐lactate‐metabolizing photosynthetic bacterium, LAB‐1, for its role in reprogramming the TIM.^[^ ^8^ ^]^ This targeted approach significantly enhanced the effectiveness of tumor immunotherapy. The development of reprogramming TIM strategies with clinical translational potential remains to be explored. Furthermore, a nanosystem combining photothermal therapy with immunomodulation represents a significant advancement in TIM reprogramming.^[^ ^9^ ^]^ However, the biosafety of this nanosystem warrants further investigation. Therefore, there is an immediate and pressing need for more effective strategies with greater clinical translational value to reprogram the TIM and increase the efficacy of immunotherapy for TNBC.

Antibody‒drug conjugates (ADCs) have emerged as novel targeted therapeutic approaches, showing substantial clinical benefits in the treatment of various solid tumors, including colorectal,^[^ ^10^ ^]^ breast,^[^ ^11^ ^]^ ovarian,^[^ ^12^ ^]^ pancreatic,^[^ ^13^ ^]^ and non‐small cell lung cancers.^[^ ^14^ ^]^ ADCs contain peptide linkers that conjugate cytotoxic drugs with tumor‐specific antibodies, facilitating the active targeting of tumors while minimizing exposure to normal tissues. These conjugates can recognize specific transmembrane proteins, internalize them into cells through receptor‐mediated endocytosis, and release cytotoxic drugs at the targeted site. The interaction between ADCs and the TIM is a multifaceted process involving various cellular and molecular mechanisms. This interaction is influenced by factors including the type and density of antigen expressed on cancer cells, the nature of the cytotoxic payload, the stability and specificity of ADCs, and the presence of immunosuppressive cells within the TIM. Understanding these interactions is crucial for developing more effective ADCs and combination therapies that can enhance the efficacy of TNBC treatment by reprogramming the TIM.

Intercellular adhesion molecule 1 (ICAM‐1), a member of the immunoglobulin superfamily, is overexpressed in various tumors,^[^ ^15^ ^]^ including TNBC, and is closely associated with metastasis and clinical prognosis. Unlike other proteins, ICAM‐1 overexpression in TNBC positively correlates with immunotherapy efficacy.^[^ ^16^ ^]^ Recent studies revealed that the expression levels of ICAM‐1 in murine models of melanoma and colon cancer were significantly correlated with the therapeutic efficacy of immune checkpoint inhibitors,^[^ ^17^ ^]^ suggesting a potential synergistic effect between these immunotherapeutic strategies, potentially leading to improved therapeutic outcomes.

In addition, B7‐H3, highly expressed in various human tumor types, has been identified as a potent immune checkpoint target.^[^ ^18^, ^19^ ^]^ B7‐H3 overexpression is linked to increased clinical tumor burden and reduced survival rates in several cancers.^[^ ^20^, ^21^ ^]^ Research has indicated that B7‐H3 might promote tumor proliferation and drug resistance by upregulating or activating various protumor signaling cascades.^[^ ^22^ ^]^ Furthermore, the B7‐H3 molecule can shape the immunosuppressive tumor microenvironment by promoting the secretion of the immunosuppressive cytokine IL‐10, thereby inhibiting T‐cell proliferation.^[^ ^23^ ^]^ The humanized antibody enoblituzumab, which targets B7‐H3, could enhance antibody‐dependent cellular cytotoxicity against a broad spectrum of tumors.^[^ ^24^ ^]^ Previous studies have demonstrated that activated T cells equipped with anti‐CD3× anti‐B7‐H3 components could effectively exert cytotoxic effects on tumor cells.^[^ ^25^ ^]^ EX105, a human bispecific antibody that targets B7‐H3 and CD3, has been approved for clinical trials in treating advanced solid malignancies due to its significant clinical translational potential.^[^ ^26^ ^]^ In addition, TAK‐280, an innovative B7‐H3× CD3ε bispecific antibody construct designed for conditional activation that strategically redirects and activates T cells, has been approved for a phase 1/2 clinical trial targeting locally advanced or metastatic solid tumors (NCT05220098).^[^ ^27^ ^]^

**Scheme 1:**
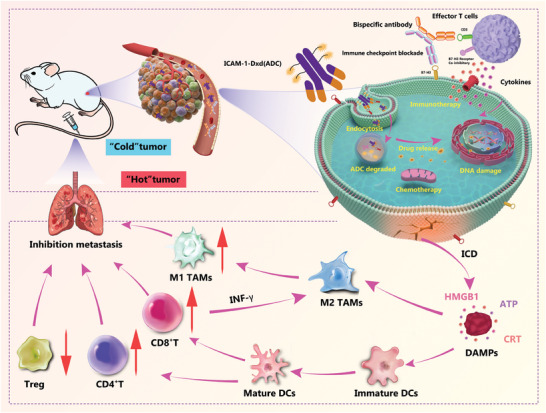
Schematic illustration of ICAM‐1‐targeted antibody–drug conjugates combined with B7‐H3‐CD3 bispecific antibodies for concurrent immunotherapy and targeted therapy to reprogram the tumor immune microenvironment to improve the efficacy of immunotherapy.

Consequently, we hypothesized combining ICAM‐1‐targeted ADCs with a B7‐H3‐CD3 bispecific antibody might elicit a synergistic antitumor effect by reprogramming the TIM in TNBC. To test this hypothesis, we conducted a proof‐of‐concept study and found that combining ICAM‐1–Dxd with a B7‐H3‐CD3 bispecific antibody effectively and persistently inhibited the in vivo growth and metastasis of TNBC by reprogramming the TIM (**Scheme** **1**).

## 2 Results

### 2.1 Assessment of ICAM‐1 Protein Expression

**Figure 1:**
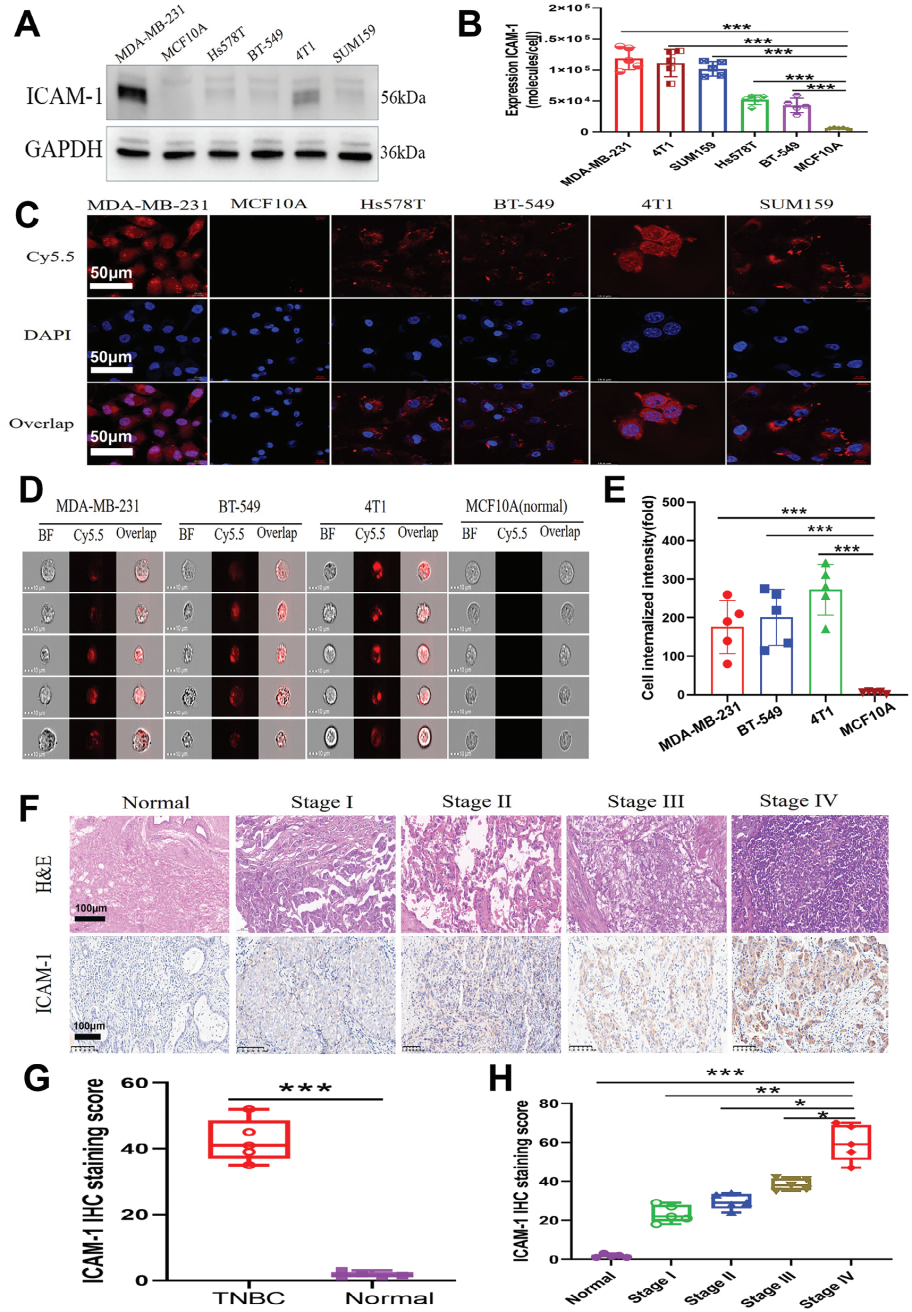
Differential overexpression of ICAM‐1 in TNBC tissues and cells. A) Western blotting of ICAM‐1 and GAPDH in TNBC cell lines. B) Flow cytometry of ICAM‐1 in TNBC cell lines. C) Cellular immunofluorescence staining. D) Antibody internalization assessment via flow cytometry imaging. E) Assessment of signal intensity for cellular internalization (*n* = 10 000). F) Representative images of IHC staining. G) Assessment of the ICAM‐1 IHC staining score. H) Correlation of tumor pathological score with TNM stage. B,E,G, and H): Results were based on five independent experiments (*n* = 5) and presented as mean ± SD, statistical significance was evaluated using an un‐paired two‐tailed *t*‐test (*p < 0.05, **p < 0.01, ***p <0.001).

ICAM‐1 protein expression was assessed in four human TNBC cell lines (MDA‐MB‐231, SUM159, Hs578T, and BT‐549) and a single established mouse TNBC cell line (4T1), with MCF10A serving as the control. Elevated ICAM‐1 expression was detected in TNBC cells via Western blot analysis, whereas ICAM‐1 was practically undetectable in MCF10A human breast epithelial cells (**Figure** **1**A; Figures S1, S2, Supporting Information). Concordant outcomes were observed via flow cytometry (Figure 1B). Additional verification of ICAM‐1 expression upregulation in TNBC cells was achieved through immunofluorescence. ICAM‐1 expression was notably elevated on the plasma membranes of TNBC cell lines, including MDA‐MB‐231, Hs578T, BT‐549, 4T1, and SUM159 (Figure 1C). By contrast, ICAM‐1 was undetectable in normal MCF10A cells. Elevated ICAM‐1 expression levels in TNBC cells facilitate straightforward assessment of treatments targeting ICAM‐1.

Recognizing the pivotal role of receptor‐mediated endocytosis in the development of ADCs, we conducted an imaging flow cytometry study to investigate this process in TNBC cells. Our findings revealed that ICAM‐1–Cy5.5 was substantially internalized by MDA‐MB‐231, SUM159, and 4T1 cells through a mechanism involving ICAM‐1 receptor‐mediated endocytosis. By contrast, ICAM‐1–Cy5.5 was scarcely detectable in MCF10A cells, which was attributed to the significant lack of ICAM‐1 receptor expression, as illustrated in Figure 1D. Compared with that in MCF10A cells, the fluorescence intensity of ICAM‐1–Cy5.5 in human TNBC cells was approximately 200 times greater (Figure 1E). These findings suggest that the ICAM‐1 antibody could be an attractive ligand for targeting TNBC tumors. To ascertain the clinical relevance of ICAM‐1 expression in TNBC tissues, we conducted IHC staining for ICAM‐1 on a series of 40 human TNBC tumor tissue samples and compared them with 40 normal mammary tissue samples. Figure 1F,G demonstrates robust overexpression of ICAM‐1 on the plasma membrane of TNBC cells derived from tumors across various pathological stages, in contrast to the minimal expression observed in normal mammary tissue. The IHC results revealed a positive correlation between the expression levels of ICAM‐1 and the TNM stage of the tumor (Figure 1H).

### 2.2 Characterization and Cytotoxicity of ICAM‐1–Dxd

**Figure 2:**
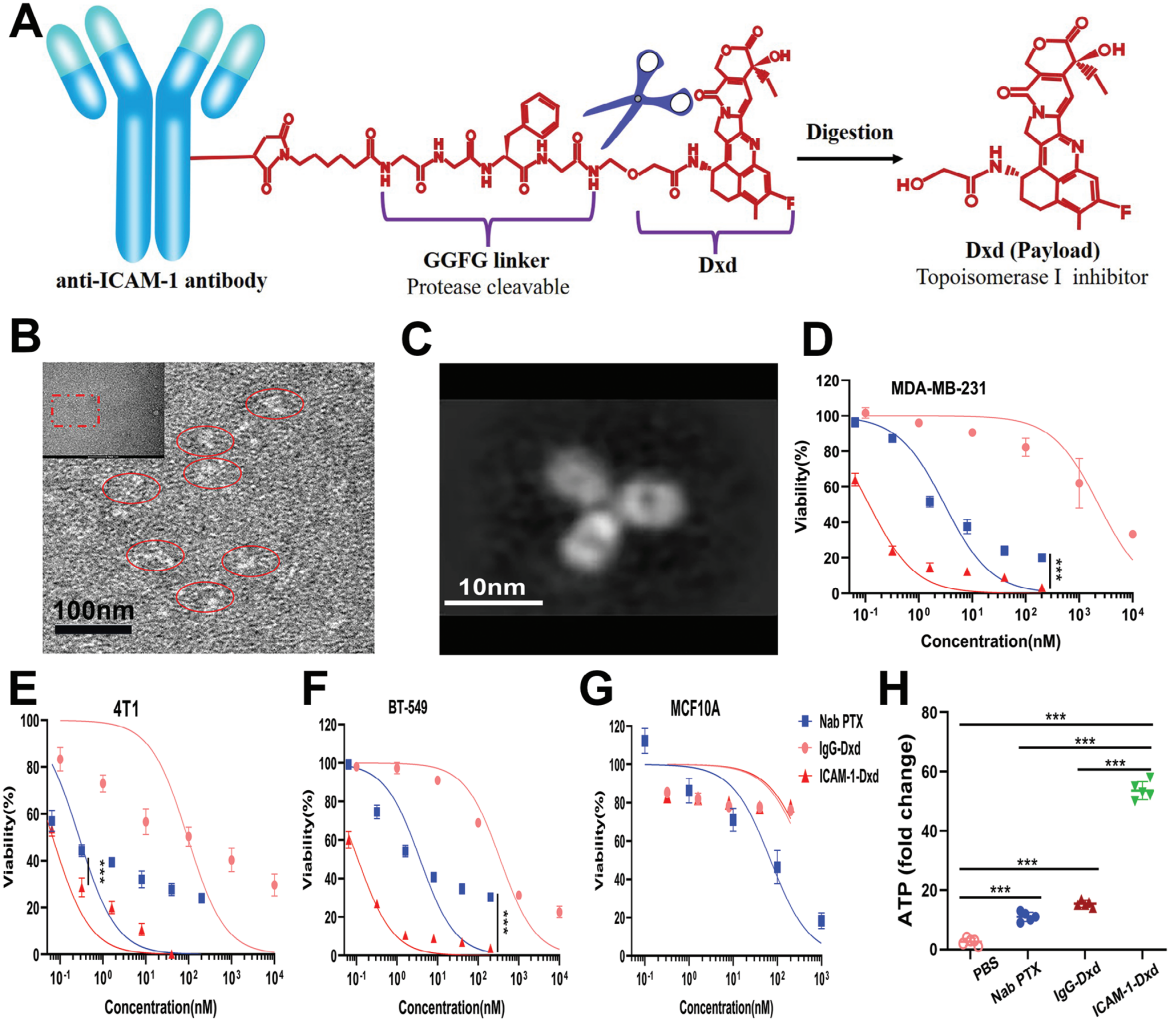
Structure and ICAM‐1‐specific activity of the ICAM‐1‐Dxd ADCs. A) Schematic structure of ICAM‐1‐Dxd. B) TEM images of negatively stained ICAM‐1‐Dxd (white dots). C) The morphological structure of ICAM‐1‐Dxd is presented using a two‐dimensional classification method. D–G) Cell viability assays to measure the antitumor effects of ICAM‐1‐Dxd on MDA‐MB‐231 D), 4T1 E), BT‐549 F), and MCF10A G) cells compared with those of IgG‐Dxd and Nab PTX. H) Release of adenosine triphosphate (ATP). D,E,F, and G): Results were based on three independent experiments (*n* = 3) and presented as mean ± SD, H): Results were based on five independent experiments (*n* = 5) and presented as mean ± S.D., statistical significance was evaluated using an un‐paired two‐tailed *t*‐test (*p < 0.05, **p < 0.01, ***p < 0.001).

To confirm the description of ICAM‐1–Dxd (Figures S3–S5, Supporting Information), its structural form and dimensions were assessed via transmission electron microscopy (TEM; **Figure** **2**B,C).

We assessed the cytotoxic effects of ICAM‐1–Dxd in TNBC cell lines (MDA‐MB‐231, 4T1, and BT‐549) and MCF10A cells.Albumin‐bound paclitaxel (Nab PTX) and nontargeting IgG–Dxd served as comparative controls.

ICAM‐1–Dxd had significant cytotoxic effects on TNBC cell lines (MDA‐MB‐231, 4T1, and BT‐549) (Figure 2D–F). The IC50 values for ICAM‐1–Dxd were 0.132 × 10^−9^ m for MDA‐MB‐231 cells, 0.075 × 10^−9^ m for 4T1 cells, and 0.141 × 10^−9^ m for BT‐549 cells. These values were markedly lower than those of Nab PTX and IgG‐DX8951, which ranged from 0.65 × 10^−9^ to 3752.6 × 10^−9^ m, indicating a greater potency of ICAM‐1–Dxd in inducing cytotoxicity.

In addition, ICAM‐1–Dxd did not have any cytotoxic effects on MCF10A cells, potentially attributable to the lack of ICAM‐1 receptor expression in these cells. (Figure 2G). Cell membrane damage induced by ICAM‐1–Dxd can release cellular contents. As shown in Figure 2H, compared with the other treatments, ICAM‐1–Dxd significantly stimulated the release of adenosine triphosphate (ATP). The results of the cytotoxicity experiments strongly support the evaluation of the tumor growth inhibition rate of ICAM‐1–Dxd in TNBC model mice.

### 2.3 TNBC Organoid Research

**Figure 3:**
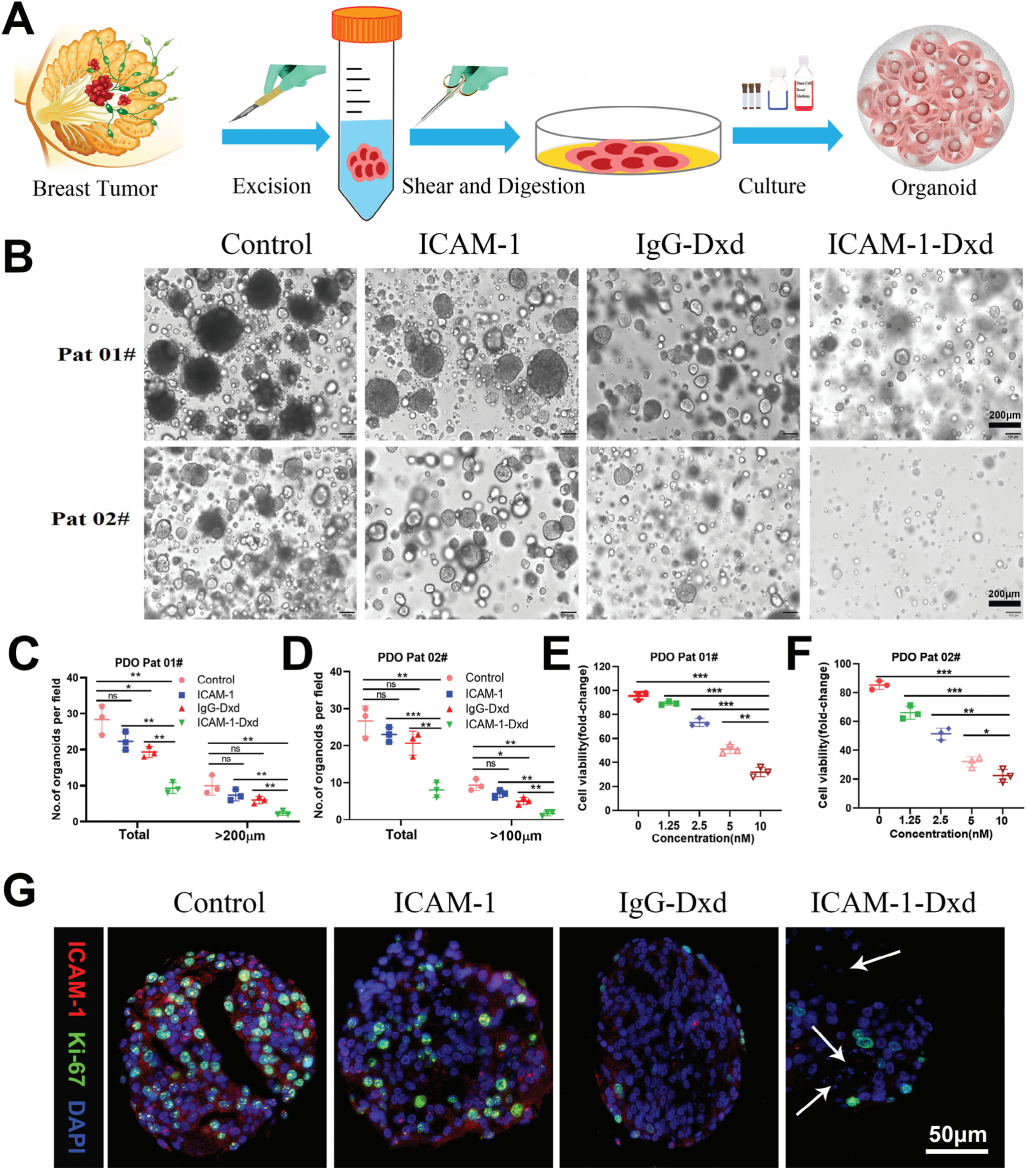
Assessment of ICAM‐1‐Dxd efficacy in patient‐derived organoid (PDO) models of TNBC. A) Schematic of the TNBC PDO model. B) Representative images of organoids from patient #1 and patient #2 across various treatment groups. C,D) Statistical analysis of TNBC PDO size and the relative number of PDOs formed. E,F) Proliferation assays of TNBC PDOs exposed to vehicle control or increasing concentrations of ICAM‐1‐Dxd. G) Immunofluorescence staining of ICAM‐1 and Ki‐67 in PDOs. The arrows denote regions of nuclear fragmentation. C), D), E) and F): Results were based on three independent experiments (*n* = 3) and presented as mean ± SD, statistical significance was evaluated using an un‐paired two‐tailed *t*‐test (*p < 0.05, **p < 0.01, ***p < 0.001).

We established a patient‐derived organoid (PDO) model utilizing TNBC specimens to further evaluate the antitumor efficacy of ICAM‐1–Dxd. Tumor cells from two patients with TNBC were isolated and cultured in a 3D system to form tumor organoids (**Figure** **3**B). Figure 3C,D shows that ICAM‐1‐Dxd markedly suppressed TNBC organoid growth, as characterized by a reduction in the organoids’ size and quantity. The dose‐dependent inhibition of growth in TNBC PDOs, as depicted in Figure 3E,F, demonstrated the robust inhibitory impact of ICAM‐1–Dxd on the progression of TNBC. Immunofluorescence experiments revealed that ICAM‐1–Dxd induced alterations in normal cellular architecture and nuclear fragmentation, indicative of cell death. However, no significant morphological changes were observed in the organoids from the other treatment groups. In addition, although ICAM‐1^+^/Ki67^+^ cancer cells survived ICAM‐1 treatment, they became exceedingly rare following ICAM‐1–Dxd treatment in organoid cultures (Figure 3G), further validating the promising therapeutic potential of ICAM‐1–Dxd in the management of TNBC.

### 2.4 Recognition and Targeting of ICAM‐1 Antibodies

**Figure 4:**
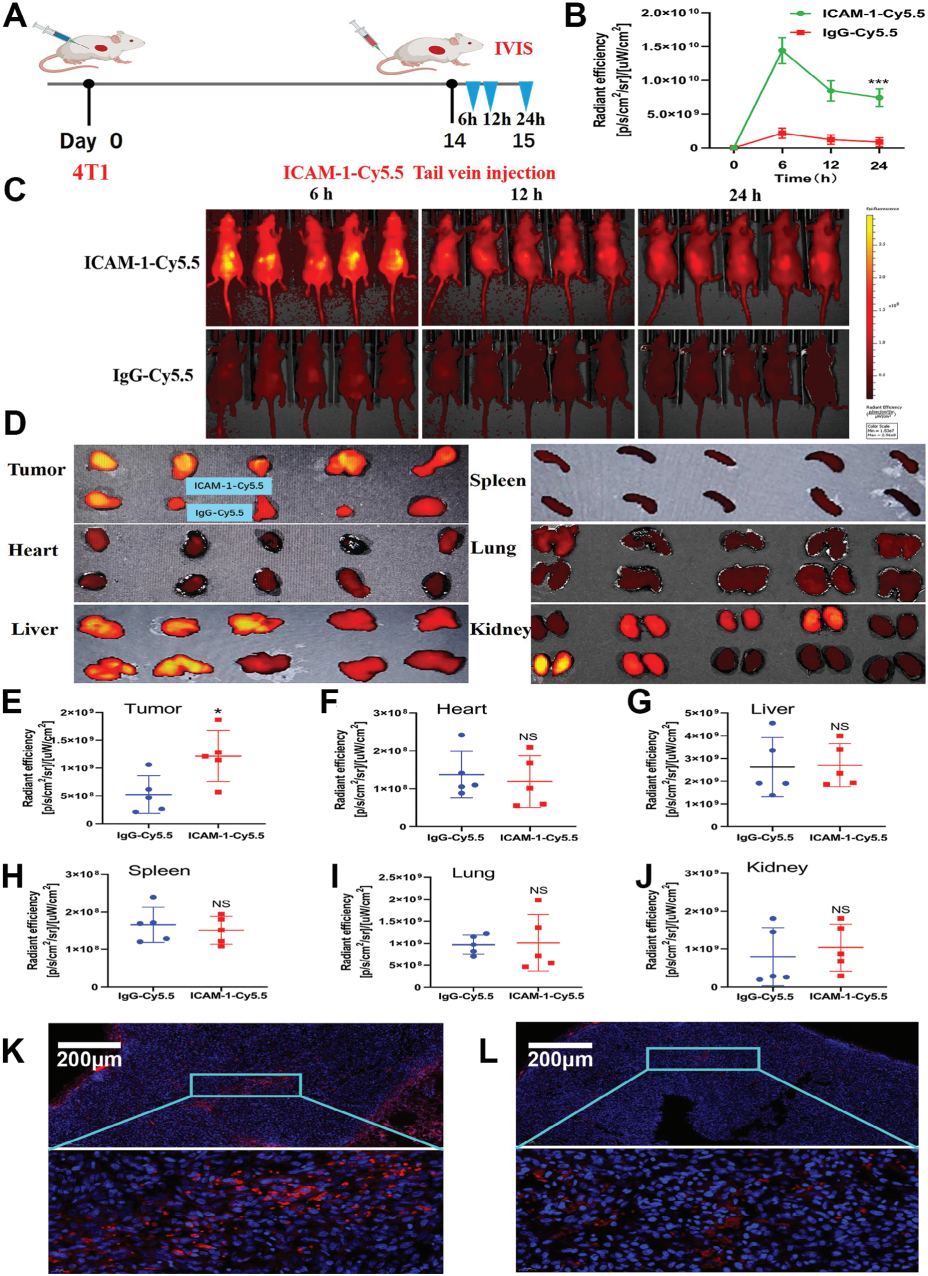
Specific recognition and biodistribution of ICAM‐1 antibodies. A) Schematic design of human TNBC biodistribution in mouse models. B) Quantification of the accumulation of IgG–Cy5.5 or ICAM‐1–Cy5.5 in 4T1 tumors. C) In vivo NIR fluorescence imaging of model mice at 6, 12, or 24 h after intravenous injection of IgG–Cy5.5 or ICAM‐1–Cy5.5 (*n* = 5). D) Ex vivo NIR fluorescence images of 4T1 tumors and five major organs treated with IgG–Cy5.5 or ICAM‐1–Cy5.5 (*n* = 5). E–J) Quantification of fluorescence intensity in 4T1 tumors and major organ accumulation of IgG–Cy5.5 or ICAM‐1–Cy5.5. K and L) Fluorescence imaging of tumor tissue sections after IgG–Cy5.5 or ICAM‐1–Cy5.5 injection. B, E, F, G, H, I and J): Results were based on five independent experiments (*n* = 5) and presented as mean ± SD, statistical significance was evaluated using an un‐paired two‐tailed *t*‐test (*p < 0.05, **p < 0.01, ***p < 0.001).

We evaluated the targeting specificity and biodistribution of ICAM‐1‐directed ADCs in 4T1 tumor‐bearing model mice (**Figure** **4**A). For this purpose, we covalently coupled ICAM‐1 mAbs with Cy5.5 (ICAM‐1–Cy5.5) (Figures S6–S8, Supporting Information) and then intravenously injected ICAM‐1–Cy5.5 into 4T1 tumor‐bearing mice at a dosage of 5 mg kg^−1^. As a control, we utilized Cy5.5‐labeled nontargeting IgG mAbs (IgG–Cy5.5).

To assess the specific accumulation of ICAM‐1–Cy5.5 antibodies in vivo, we quantified fluorescence intensities at various time intervals following injection (6, 12, and 24 h) via an IVIS spectrum imaging system. Figure 4B shows that the fluorescence intensity of the tumor region in the mice treated with ICAM‐1–Cy5.5 was markedly greater than that in the IgG–Cy5.5 control group at all the measured time points, confirming the specific targeting of tumor tissue by the ICAM‐1–Cy5.5 antibodies. In vivo, near infrared fluorescence imaging of the model mice was performed at different time points (Figure 4C). The biodistribution profile of ICAM‐1–Cy5.5 was further evaluated across five major organs and tumor sites (Figure 4D). A significantly increased accumulation of ICAM‐1–Cy5.5 was observed within the tumor tissues (Figure 4E). Specific agglomeration of ICAM‐1–Cy5.5 was not detected in the heart, liver, spleen, lung, or kidney (Figure 4F–J; Figures S9–S12, Supporting Information). Conversely, the targeted accumulation of ICAM‐1–Cy5.5 in the tumor region was clearly demonstrated through fluorescence microscopy examination of tumor tissue sections (Figure 4K,L). These in vivo findings also contributed significantly to the evaluation of the antitumor efficacy of ICAM‐1–Dxd in TNBC mouse models.

### 2.5 Pharmacokinetics of ICAM‐1‐Dxd

To assess the pharmacokinetics of ICAM‐1‐Dxd, we employed the ELISA technique to quantify the levels of total antibodies and the conjugated Dxd (ADC) in the serum of healthy mice following intravenous administration through the tail vein. Our findings revealed that the PK profiles of both total antibody and conjugated Dxd exhibited a pattern of gradual decline over the course of repeated dosing (three cycles) (Figures S13, S14, Supporting Information). According to the Winnonlin software analysis, the elimination half‐lives for the total antibody and conjugated Dxd were 148 and 139 h, during the initial phase (days 1–7), and 161 and 168 h, during the subsequent phase (days 15–21), respectively (Tables S1, S2, Supporting Information).

### 2.6 Bioinformatics Analysis of the B7‐H3 Gene

#### 2.6.1 Data Collection

The TCGA‐TNBC dataset comprised 113 adjacent tissue samples and 103 TNBC samples. The differences between the TNBC and normal groups in this dataset were analyzed. According to the median expression of the B7‐H3 gene, TNBC patients were divided into high‐ and low‐expression groups for differential analysis. The R package “DESeq2” was used to analyze the differences between the two sets of samples (TNBC vs control and high vs low). |log2FC| > 2 and p.adj < 0.05 were used as screening criteria to select differentially expressed genes. There were 2051 DEGs between the TNBC and control groups, of which 1145 demonstrated upregulated expression in the disease group and 906 demonstrated downregulated expression. There were 119 DEGs2 in the B7‐H3 high‐low‐expression group (high vs low). In this group, 82 demonstrated upregulated expression, and 37 downregulated expression. To obtain a more intuitive observation of genetic differences, we used the R package “ggplot2.” Volcano of the differentially expressed genes were constructed, and the top 10 upregulated genes (log2FC) were selected. We obtained 2051 DEGs in the control group and 2 DEGs in the 119 B7‐H3 high‐low‐expression groups, revealing the DEGs shared between the two groups. A total of 51 intersecting genes were identified and recorded as candidate genes in the subsequent analysis (Figure S15, Supporting Information).

#### 2.6.2 Analysis of B7‐H3

**Figure 5:**
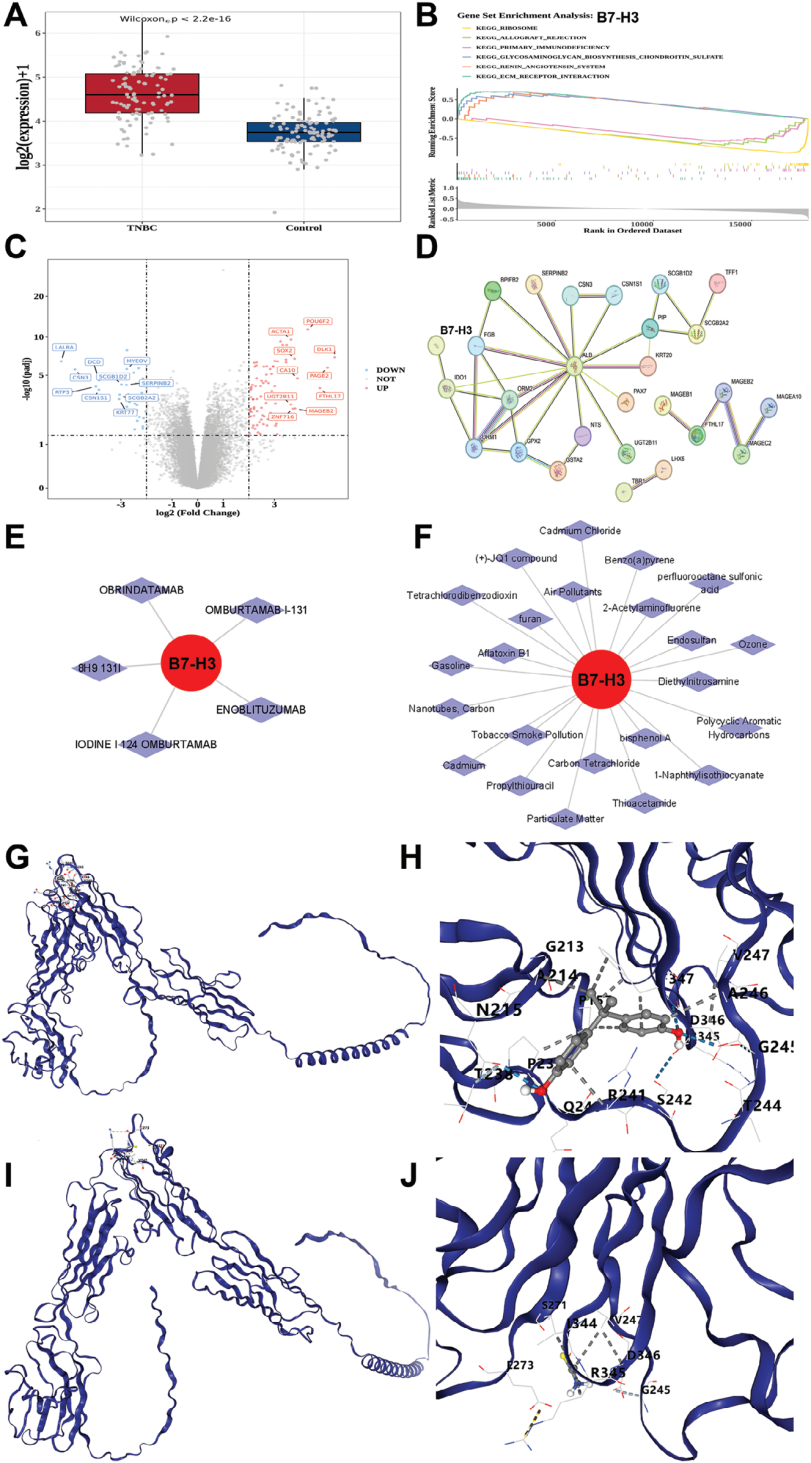
The biological functions of B7‐H3 were analyzed via bioinformatic analysis. A) Differential expression of B7‐H3 between cancer and normal tissues in the TCGA‐TNBC database. B) Gene set enrichment analysis of the B7‐H3 pathway. C) Volcanic of the expression of differentially expressed genes in B7‐H3 (high versus low). D) B7‐H3‐related protein‒protein interaction (PPI) signaling pathways. E) Potential drugs targeted by B7‐H3. F) Screening of small molecule compounds that interact with B7‐H3. G) Molecular docking study of bisphenol A and B7‐H3. H) Magnified image of the binding sites of bisphenol A and B7‐H3. I) Molecular docking study of thioacetamide and B7‐H3. J) Magnified image of the binding sites of thioacetamide and B7‐H3. The blue ribbon represents the B7‐H3 protein molecule, the gray stick model represents the small molecule compound, the blue dashed lines represent hydrogen bonds, the gray dashed lines represent hydrophobic interactions, and the yellow dashed lines represent ionic interactions.

Our results demonstrated that the expression of B7‐H3 was significantly greater in the TNBC group than in the control group (**Figure** **5**A). In the GSEA, B7‐H3 was enriched in 69 pathways, with the top three positively and negatively enriched pathways (Figure 5B). A volcano map (Figure 5C) of the DEGs was constructed, and the top 10 upregulated genes (log2FC) were selected. The number of DEGs was significantly greater in the TNBC group versus the control group (Figures S16, S17, Supporting Information). GO enrichment analysis revealed 148 differences (Figure S18, Supporting Information).

#### 2.6.3 Protein‐protein interactions (PPI) of B7‐H3

To better understand the interactions among the candidate genes, we introduced 52 candidate genes into STRING to construct a PPI network with a confidence equal to 0.4, with the interaction network among candidate genes depicted in Figure 5D. After the 25 discrete genes were excluded, the network diagram included 27 nodes and 36 edges. Among these, B7‐H3 was found to interact with IDO1.

#### 2.6.4 Drug Prediction

To explore potential therapeutic agents for TNBC, we conducted drug screening against the target B7‐H3, which yielded five candidate drugs. The mRNA drug network used in this study was constructed via Cytoscape Soft (Figure 5E). On the basis of the predicted drugs, we utilized combination therapy with a bispecific antibody targeting B7‐H3 and CD3 (obrindatamab) to improve the efficacy of immunotherapy for TNBC.

#### 2.6.5 Compound Prediction and Molecular Docking

The CTD database (https://ctdbase.org/) was used to identify the compounds related to B7‐H3. On the basis of the number of interactions, bisphenol A, particulate matter, and thioacetamide were selected as target compounds (Figure 5F).

Drugs with high B7‐H3 scores in the CTD database were selected for the molecular docking analysis. As the thioacetamide particulate matter did not correspond to the three‐dimensional structure, the final docking relationship pair included B7‐H3‐bisphenol A and B7‐H3‐thioacetamide. A three‐dimensional structural database of the drugs was subsequently obtained. Two protein structure pairs predicted by B7‐H3 were obtained from the UniProt database (https://www.uniprot.org/org/). The relationship pairs were subjected to molecular docking analysis. A docking fraction less than –1.2 kcal mol^−1^ indicates that the selected drug has a high binding affinity for the target. The docking fractions of B7‐H3‐bisphenol A (Figure 5G,H) and B7‐H3‐thioacetamide (Figure 5I,J) were –6.8 and –2.4 kcal mol^−1^, respectively. The docking fractions were all lower than –1.2 kcal mol^−1^, indicating a high affinity between small‐molecule compounds and their corresponding biomarkers. Thus, specific drugs targeting the B7‐H3 antigen warrant further investigation.

### 2.7 Antitumor Efficacy of ICAM‐1–Dxd Plus B7‐H3‐CD3 in 4T1 Xenograft Models

To determine the optimal dosage of ICAM‐1‐Dxd, its antitumor efficacy was evaluated in 4T1 tumor‐bearing mice model using escalating doses (1, 5, or 10 mg kg^−1^) administered via tail vein injection. The tumor growth inhibition rates for ICAM‐1‐Dxd at dosages of 1, 5, and 10 mg kg^−1^ were 51.3%, 93.6%, and 97.8% greater, respectively, compared to the PBS control group (Figure S19‐S21, Supporting Information). The findings indicated that a dosage of 5 mg kg^−1^ ICAM‐1‐Dxd significantly and sustainably suppressed the growth of TNBC tumors. Furthermore, no significant differences in mouse weight were observed across the various dosage groups (Figure S22, Supporting Information). Therefore, the 5 mg kg^−1^ dosage was identified as the optimal concentration of ICAM‐1‐Dxd for targeted therapy of TNBC in preclinical mouse models.

To further confirm the expression of the B7‐H3 protein in 4T1 cells, we employed immunofluorescence methods. The expression levels of B7‐H3 in the 4T1 cells were significantly greater than those in the control cells (MCF10A) (Figure S23, Supporting Information).

**Figure 6:**
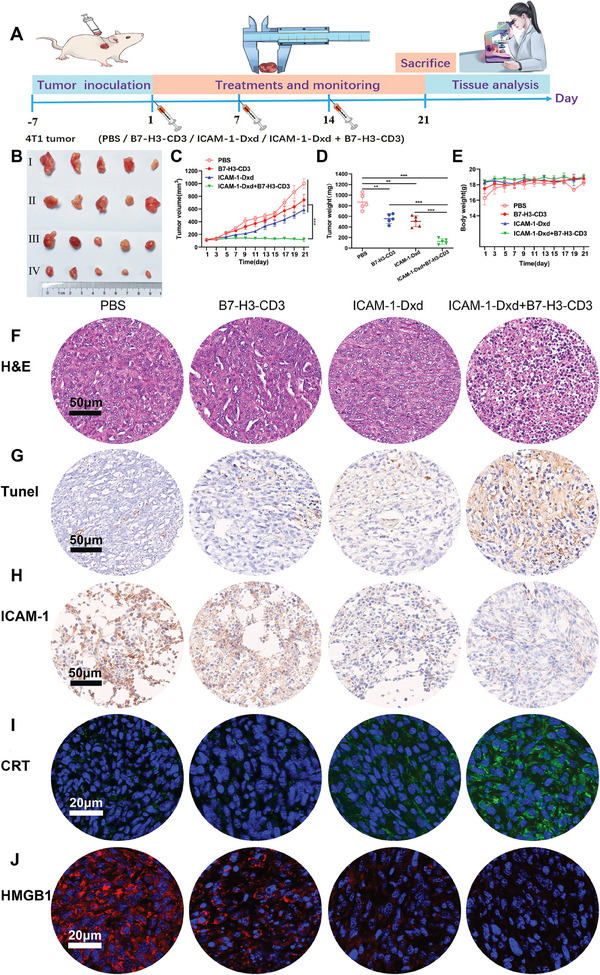
Antitumor activity of ICAM‐1–Dxd plus B7‐H3‐CD3 in 4T1 TNBC tumors in vivo. A) Schematic design of in vivo efficacy in TNBC mouse models. B) Ex vivo mouse tumor imaging (*n* = 5). C) Changes in the volume of the tumors. D) The tumor weights across different groups. E) Curve of weight change in the mice. F–H) Representative images of H&E staining, TUNEL staining, and ICAM‐1 expression. I and J) Fluorescence images of CRT (I) exposure and HMGB1 (J) secretion in tumor sections from each group of mice. C, D, and E): Results were based on five independent experiments (*n* = 5) and presented as mean ± SD, statistical significance was evaluated using an un‐paired two‐tailed *t*‐test (*p < 0.05, **p < 0.01, ***p < 0.001).

We evaluated the efficacy of combination therapy involving ICAM‐1–Dxd and B7‐H3‐CD3 bispecific antibodies in a TNBC model (4T1) (**Figure** **6**A). Compared with PBS, B7‐H3‐CD3 (5 mg kg^−1^), or ICAM‐1–Dxd (5 mg kg^−1^) (Figure 6B,D), the combination of ICAM‐1–Dxd and B7‐H3‐CD3 resulted in significant and sustained inhibition of tumor growth in tumor‐bearing model mice. The weight of the tumors in the combination therapy group was 86.71% greater than that in the PBS group (Figure 6D). The data indicated that the combination therapy of ICAM‐1–Dxd plus B7‐H3‐CD3 elicited significant antitumor activity in 4T1 tumors compared with the groups treated with B7‐H3‐CD3 or ICAM‐1–Dxd alone. The enhanced antitumor efficacy was likely attributed to the immunogenic cell death (ICD) induced by the combination therapy. The antitumor mechanism of the combination therapy was further detected through Terminal dUTP Nick End Labeling (TUNEL) and ICAM‐1 staining methods. TUNEL staining increased and ICAM‐1 expression decreased in the ICAM‐1–Dxd plus B7‐H3‐CD3 group compared with the other groups (Figure 6G,H).

ICD can effectively stimulate the release of molecular damage molecular patterns (DAMPs), including calreticulin (CRT) and high‐mobility group box 1 (HMGB1).^[^ ^31^ ^]^ The effects of exposure to CRT and the migration of HMGB1 in tumor tissues were explored via immunofluorescence staining (Figure 6J,K). More exposed CRT (green fluorescence) was detected on the cell membranes after combination therapy with ICAM‐1–Dxd and B7‐H3‐CD3 (Figure 6I). Intense red fluorescence (HMGB1) that overlapped with the nucleus was observed in the control group, whereas red fluorescence was almost absent in the combination therapy group (Figure 6J). These results unequivocally demonstrated that the combination therapy induced extensive ICD. Surface‐exposed CRT emits an “eat‐me” signal,^[^ ^32^ ^]^ which activates antigen‐presenting cells to phagocytose debris and remnants of dying tumor cells. In addition, migratory HMGB1 is considered a natural adjuvant that promotes DC maturation, leading to T‐cell activation. These findings provide the preconditions for stimulating the immune response in vivo.

There were no significant differences in mouse body weight across the treatment groups (Figure 6E). The in vivo toxicity of ICAM‐1–Dxd and B7‐H3‐CD3 combination therapy was also evaluated by serum analysis. Briefly, mouse blood samples were collected and analyzed to determine the levels of aspartate aminotransferase (AST) and alanine aminotransferase (ALT). The levels of AST and ALT in the combination therapy group were not elevated compared with those in the control group. In addition, renal toxicity was evaluated by monitoring changes in creatinine and blood urea nitrogen (BUN) levels, with no evidence of renal toxicity observed across the different groups (Figure S24, Supporting Information).

### 2.8 Assessment of Antitumor Immunity In Vivo

**Figure 7:**
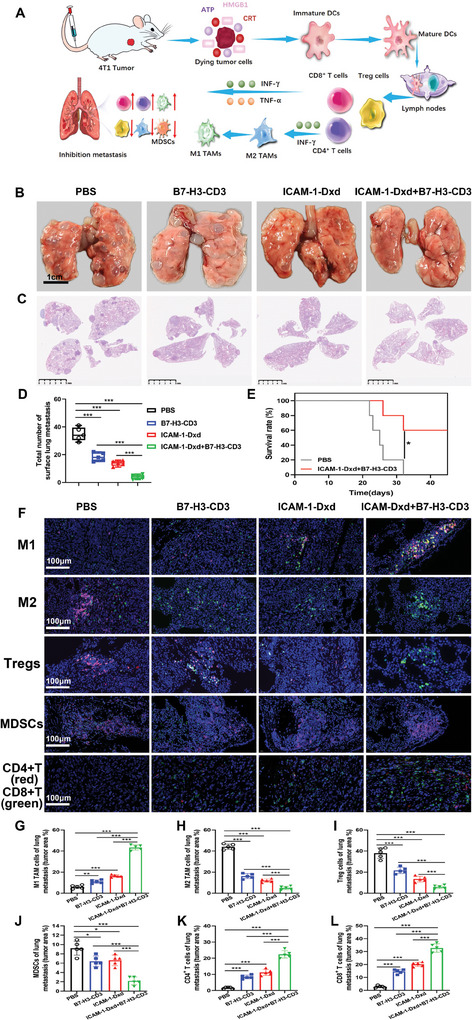
Analysis of the tumor immune microenvironment in antitumor lung metastasis. A) Schematic illustration of damage‐associated molecular patterns that induce organismal immunity. B) Representative images of lung metastases. C) Hematoxylin and eosin (H&E) staining of lung tissue. D) Counting of lung metastatic lesions. E) Survival curves of the mice (*n* = 5). F) Immunofluorescence imaging of different immune cell subpopulations. M1‐type macrophages: CD11b^+^ (red)/F4/80^+^ (green)/CD80^+^ (pink); M2‐type macrophages: CD11b^+^ (red)/F4/80^+^ (green)/CD206^+^ (pink); Tregs: CD3^+^ (red)/CD4^+^ (green)/Foxp3^+^ (pink); and MDSCs: CD45^+^ (red)/CD11b^+^ (pink)/Gr‐1^+^ (green). G–L) Quantitative analysis of M1‐type macrophages G), M2‐type macrophages H), Tregs I), MDSCs J), CD4^+^ T cells K), and CD8^+^ T cells L) in lung metastatic lesions. D, E, G, H, I, J, K, and L): Results were based on five independent experiments (*n* = 5) and presented as mean ± SD, statistical significance was evaluated using an un‐paired two‐tailed *t*‐test (*p <0.05, **p < 0.01, ***p < 0.001).

We next assessed the potential of combination therapy to combat tumor metastasis. The experimental results indicated that there were very few metastatic nodules in the lung images and H&E‐stained sections from the combination therapy group versus the multiple lung metastatic lesions observed in the control group (**Figure** **7**B–D). Furthermore, compared with the PBS group, the combination therapy of ICAM‐1–Dxd with B7‐H3‐CD3 notably prolonged the survival time of the mice, underscoring the robust antimetastatic efficacy of the combined therapeutic approach (Figure 7E).

M2‐type tumor‐associated macrophages (TAMs) are a subpopulation of macrophages with immunosuppressive properties that can inhibit T‐cell proliferation.^[^ ^33^ ^]^ M1‐type TAMs are key in immune defense and inflammatory responses.^[^ ^34^ ^]^ Consequently, promoting the repolarization of macrophages from the M2 type to the M1 type is an important direction for improving the TIM. To further explore the role of combination therapy with ICAM‐1–Dxd and B7‐H3‐CD3 in remodeling the TIM, different types of immune cells in lung metastases were identified and detected through fluorescence staining methods (Figure 7F). The proportions of M1‐ and M2‐type TAMs in lung metastatic lesions and the expression levels of correlative cytokines were analyzed. Compared with that in the control group, the percentage of M1‐type macrophages in the combined treatment group significantly increased from 6.0% to 43.3% (Figure 7G), whereas the percentage of M2‐type macrophages markedly decreased (Figure 7H). Furthermore, interleukin 10 (IL‐10), which is secreted by M2‐type TAMs and acts as an anti‐inflammatory factor, was reduced fourfold compared with that in the control group (Figure S25, Supporting Information), and IL‐12, which is secreted by M1‐type TAMs and acts as a proinflammatory factor,^[^ ^35^ ^]^ was increased by 6.7‐fold (Figure S25, Supporting Information), demonstrating that the combined treatment promoted the conversion of tumor‐promoting M2‐type TAMs to tumor‐inhibiting M1‐type TAMs. The potential of the combined treatment to activate immune responses was further assessed by investigating DC maturation in vivo. As demonstrated in Figure S26 (Supporting Information), the maturation rate of DCs in tumor‐infiltrating lymph nodes was also obviously increased by combination therapy with ICAM‐1–Dxd and B7‐H3‐CD3.

As mature DCs and bispecific antibodies (B7‐H3‐CD3) can activate effector T cells, the activation of T cells was also explored. As shown in Figure 7K,L, the counts of CD4+ and CD8+ T cells were increased by 14.0‐ and 11.7‐fold, respectively. Moreover, regulatory T cells (Tregs) are widely recognized as accomplices in tumor metastasis and immune evasion and can suppress the activity of effector T cells. As shown in Figure 7I, the percentage of Tregs was effectively reduced in the combination therapy group. In addition, myeloid‐derived suppressor cells (MDSCs) possess potent immunosuppressive activity and promote tumor growth and metastasis. Compared with that in the control group, the ratio of MDSCs in the combined treatment group was also significantly lower (Figure 7J). Collectively, the changes in immune cells after administration indicated that the TIM could be effectively reshaped. As shown in Figure S25 (Supporting Information), the levels of IL‐6, interferon‐γ (INF‐γ), and tumor necrosis factor‐α(TNF‐α), common proinflammatory cytokines, were significantly elevated in the combined treatment group. Specifically, these levels were 5.9‐fold, 3.5‐fold, and 4.4‐fold greater than those in the control group, indicating that the immune response was effectively triggered. The results revealed that the combined treatment effectively reshaped the TIM and triggered a robust and intense immune response. However, the strategy of combining the blockade of immune checkpoints, represented by B7‐H3‐CD3 bispecific antibodies, with ADCs to reprogram the TIM requires further exploration.

### 2.9 Antitumor Efficacy of ICAM‐1‐Dxd Plus B7‐H3‐CD3 in Patient Derived Xenograft (PDX) Models

**Figure 8:**
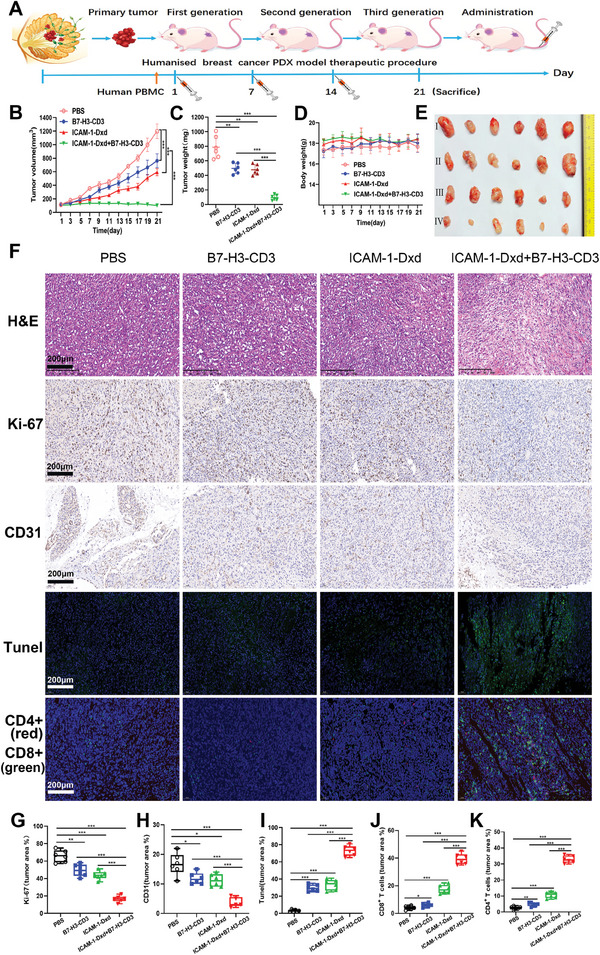
Antitumor activity of ICAM‐1–Dxd plus B7‐H3‐CD3 in TNBC PDX models. A) Schematic diagram of the TNBC PDX model, receiving PBS+PBMC, B7‐H3‐CD3+PBMC, ICAM‐1–Dxd+PBMC, or ICAM‐1–Dxd + B7‐H3‐CD3+ PBMCs across various time points (*n* = 6). B) Volume changes in the tumors. C) The tumor weights across different groups. D) Curve of weight change in the mice. E) Imaging of dissected tumor tissues. F) Histological analysis of tumor sections. G–K) Quantitative analysis of Ki67 G), CD31H), TUNEL (I), CD4^+^ T J), and CD8^+^ T K) cells in tumors. B, C, D, G, H, I, J, and K): Results were based on six independent experiments (*n* = 6) and presented as mean ± SD, statistical significance was evaluated using an un‐paired two‐tailed *t*‐test (*p < 0.05, **p < 0.01, ***p < 0.001).

Next, we evaluated the antitumor effects of ICAM‐1‐Dxd plus B7‐H3‐CD3 in PDX TNBC tumor models (**Figure** **8**A). ICAM‐1‐Dxd was intravenously administered to the PDX xenograft models at 5 mg kg^−1^ once a week for 3 weeks. B7‐H3‐CD3 bispecific antibody was administered intraperitoneally at a dose of 5 mg kg^−1^. After 21 days, treatment with ICAM–Dxd combined with B7‐H3‐CD3 resulted in significant and sustained antitumor activity, outperforming the other groups (Figure 8B,C). Quantitative analysis of the tumor mass revealed that combination therapy with ICAM‐1–Dxd and B7‐H3‐CD3 led to a reduction in TNBC tumor growth compared with that in the control group (Figure 8E). The most pronounced induction of apoptosis was observed in the group treated with the combination of ICAM‐1–Dxd and B7‐H3‐CD3 (Figure 8F). In addition, compared with the other groups, the ICAM‐1–Dxd plus B7‐H3‐CD3 group presented a significantly reduced number of Ki67‐positive cells, leading to substantial and sustained inhibition of tumor growth (Figure 8G). Moreover, decreases in CD31 expression levels and increases in green fluorescence in TUNEL staining were observed in the ICAM‐1–Dxd plus B7‐H3‐CD3 group compared with the other groups (Figure 8H,I). Importantly, the percentage of CD8+ and CD4+ T cells in the ICAM‐1–Dxd plus B7‐H3‐CD3 group was markedly greater than that in the other groups (Figure 8J,K), demonstrating immune cell infiltration in tumor tissues.

Finally, we investigated the antitumor immune responses triggered by this synergistic treatment to further ascertain the potent antitumor effects of ICAM‐1–Dxd in combination with B7‐H3‐CD3. Compared with other treatments, combination therapy with ICAM‐1–Dxd and B7‐H3‐CD3 significantly elevated the concentrations of tumor‐suppressive cytokines, including TNF‐α, IL‐6, and IFN‐γ, to a greater extent (Figure S27, Supporting Information). These results indicated that combined therapy with ICAM‐1–Dxd and B7‐H3‐CD3 could induce antitumor immune responses and markedly augment the efficacy of synergistic treatment in TNBC PDX models.

## 3 Discussion

In the field of TIM reprogramming, TNBC research progress has demonstrated that TIM plays a significant role in the occurrence and development of this disease. The TIM is a complex environment comprising tumor cells, stromal cells, the extracellular matrix, microvessels, and various signaling molecules.^[^ ^36^ ^]^ The TIM enhances tumor cells’ proliferation, invasion, metastasis, and immune escape capabilities, hindering the antitumor response in TNBC.^[^ ^37^ ^]^ Emerging studies have suggested that reprogramming the TIM to improve TNBC treatment efficacy is a promising path warranting further exploration.

We developed a targeted and potent ADC, ICAM‐1‒Dxd, and used it in combination with a B7‒H3‒CD3 bispecific antibody to achieve synergistic enhancement, demonstrating a promising strategy for reprogramming the TIM in TNBC with high clinical translational value. The results showed that cytotoxic drugs could be delivered to TNBC cells via ICAM‐1 receptor‐mediated cell membrane targeting through the conjugation of ICAM‐1 antibodies with small‐molecule drugs, which reduced the accumulation of drugs in normal tissues, increased drug specificity, and significantly minimized potential off‐target effects. Furthermore, the anti‐B7‐H3 antibody in the novel immune checkpoint inhibitor B7‐H3‐CD3 bispecific antibody specifically targeted tumor cells, not only by blocking inhibitory signaling pathways to suppress tumor immune evasion and promote T‐cell activation but also by recruiting T cells through the CD3 arm, facilitating the formation of immune synapses and reducing the spatial barrier between T cells and tumor cells, thereby activating T cells. The close‐range release of cytokines induces ICD and promotes the release of tumor antigens, which is beneficial for activating various immune cells and reshaping the TIM. Therefore, combining ICAM‐1–Dxd with a B7‐H3‐CD3 bispecific antibody might increase the efficacy of immunotherapy for TNBC through synergistic effects. The experimental results indicated that combining ICAM‐1–Dxd with the B7‐H3‐CD3 bispecific antibody effectively inhibited the growth of TNBC tumors and suppressed lung metastasis in 4T1 model mice. Moreover, in PDX mouse models, this combined treatment showed positive and encouraging antitumor activity. Our new strategy relies on ICAM‐1, identified as the best target for TNBC through quantitative screening. Furthermore, to increase the efficacy of immunotherapy for TNBC, the introduction of the B7‐H3‐CD3 bispecific antibody allowed the assessment of whether the combination of ICAM‐1–Dxd with B7‐H3‐CD3 could reprogram the TIM to reverse the tumor immune “cold” phenotype into the “hot” inflammatory phenotype.

Our data provide evidence that ICAM‐1–Dxd combined with B7‐H3‐CD3 represents a targeted and synergistic immunotherapy strategy for TNBC. Numerous strategies have been developed to reshape the TIM to increase the efficacy of immunotherapy.^[^ ^38^ ^]^ However, the biosafety and clinical translation value of some drugs warrants further investigation. Compared with drug‐loaded microsphere delivery systems with a diameter of 100 nm, ICAM‐1–Dxd ADCs have a significant size advantage (≈10–20 nm), which allows them to evade phagocytosis by the reticuloendothelial system, contributing to prolonged peripheral blood circulation.^[^ ^39^ ^]^ ICAM‐1–Dxd possesses specific active targeting capabilities, whereas most drug‐loaded microsphere delivery systems depend on passive targeting, which could lead to significant off‐target effects and potentially severe side effects.^[^ ^40^ ^]^ The advantages of active targeting and small particle size would help achieve more effective target accumulation and reduce distribution to major organs. Furthermore, several ADCs have been applied clinically to improve patient survival;^[^ ^41^ ^]^ however, few nanodrug delivery systems have been approved for clinical practice. Our novel concept of combining small‐molecule cytotoxic drugs with targeted antibodies can significantly increase the efficacy of these cytotoxic agents.

The notion of ADC as a “magic bullet,” a concept first envisioned by Paul Ehrlich in the early 20th century, is both captivating and full of potential.^[^ ^42^ ^]^ However, the realm of ADCs has faced a multitude of unforeseen hurdles and, to date, has realized only modest triumphs. A principal obstacle in the development of ADCs lies in their inherent toxicity, the majority, if not all, of the ADCs employed clinically exhibit a therapeutic index that is akin to that of the unbound chemotherapy drugs themselves. Moreover, ADCs are specific to human antigens and do not exhibit cross‐reactivity with corresponding mouse antigens, which poses a challenge when comparing on‐target and off‐target toxicities in mouse models. With the maturation of the ADC field, the issue of off‐tumor targeting toxicity is infrequently investigated during preclinical studies and is typically only tackled indirectly in later primate toxicity assessments or during Phase I clinical trials.

Our results indicated that ICAM‐1–Dxd specifically targeted TNBC, considering the overexpression of ICAM‐1 and its ideal cellular internalization. DS‐8201, an ADC approved for treating a spectrum of HER2‐positive tumor types, has demonstrated exceptional efficacy in the treatment of breast cancer. In addition, numerous ADC drugs targeting HER2‐positive breast cancer are currently under clinical investigation.^[^ ^43^ ^]^ However, regarding TNBC, the approved ADC drug TROP2‐ADC benefits only a very small number of patients owing to its severe hematological toxicity. By contrast, the protein expression level of ICAM‐1 in TNBC cells was relatively greater than that of EGFR (≈3.5 times greater than that of the control group); the protein expression level of EGFR was roughly equivalent to that of the control group. The elevated expression of ICAM‐1 in TNBC cells suggests that it could serve as an ideal and potent therapeutic target.

A previous study demonstrated that the TIM is closely related to various immunosuppressive factors, such as Tregs, MDSCs, and immunosuppressive cytokines.^[^ ^44^ ^]^ B7‐H3‐CD3 bispecific antibodies help overcome these immunosuppressive factors by activating T cells and reshaping the TIM.^[^ ^45^ ^]^ Another study characterized an IgG‐based B7‐H3‐CD3 bispecific antibody, demonstrating excellent tumor cell killing ability, T‐cell activation, proliferation, and memory formation in vitro. In three independent animal models, B7‐H3‐CD3 showed strong antitumor activity in preventing lung metastasis, inhibiting the growth of flank tumors, and eliminating large established tumors by reshaping the TIM, with no significant toxic reactions observed.^[^ ^26^ ^]^ Our research results indicate that in tumor‐bearing mouse models, combined treatment with ICAM‐1–Dxd and B7‐H3‐CD3 promotes the maturation of DCs, activates T cells, triggers the repolarization of M2‐type macrophages to M1‐type macrophages, inhibits the proliferation of Tregs and MDSCs, reprograms the TIM of TNBC, and suppresses tumor lung metastasis.

In clinical practice, combination therapy with ADC is a commonly used and promising treatment strategy. The strategy of combining ADCs with immune checkpoint inhibitors may have the following advantages: 1) Enhanced anti‐tumor activity: Sacituzumab Govitecan in combination with pembrolizumab demonstrated encouraging activity in all PD‐L1 subgroups and histologies of metastatic non‐small cell lung cancer patients studied.^[^ ^46^ ^]^ 2) Overcoming drug resistance: In HER2‐expressing metastasis breast cancer, T‐DXd plus nivolumab increased the response rate in drug‐resistant patients.^[^ ^47^ ^]^ 3) Reshaping the TIM: The combination therapy can significantly promote the infiltration of CD8+ T cells and reduce regulatory T cells.^[^ ^48^ ^]^ However, the strategy of combining ADC with immune checkpoint inhibitors also has some disadvantages: 1) Toxicity overlap: Toxicity overlap can increase adverse reactions in patients. For example, some patients may experience immune‐related adverse events such as immune‐mediated pneumonitis and hepatitis.^[^ ^49^ ^]^ 2) Drug interactions: The combination may lead to drug interactions, which can affect the efficacy and safety of the drugs. 3) Uncertainty of efficacy: Although some efficacy has been shown in some preclinical and clinical trials, more clinical data are still needed to validate the long‐term efficacy and safety of this combination therapy strategy.

Previous studies have considered ICAM‐1, a transmembrane receptor protein present in numerous solid tumors, offering promising proof‐of‐concept data on its antitumor potential. These insights motivated us to explore the synergy of ICAM‐1 antibodies with additional immunotherapeutic approaches, including immune checkpoint inhibitors. In this preclinical study, combining ICAM‐1–Dxd with B7‐H3‐CD3 exhibited synergistic antitumor effects. Although there are inevitable differences between mice and patients when these outcomes are applied in clinical practice, the combination of ICAM‐1–Dxd with B7‐H3‐CD3 could be a promising potential therapeutic strategy that leverages the synergistic antitumor effects of ADCs and immunotherapy. The antitumor effect of ICAM‐1–Dxd in combination with B7‐H3‐CD3 was superior to that of monotherapy. The possible mechanisms include 1) targeted accumulation and site‐specific release of cytotoxic drugs; 2) blocking the inhibitory signaling pathway of B7‐H3‐CD3, suppressing tumor immune evasion, improving the T‐cell response, and synergizing with ICAM‐1–Dxd to enhance efficacy; and 3) inducing immunogenic cell death, in which tumor cells produce natural tumor‐associated antigens and release DAMPs, which interact with pattern recognition receptors on the surface of DCs, activating the immune response and reprogramming the TIM. The findings from this investigation offer compelling preclinical support for combining ICAM‐1–Dxd with B7‐H3‐CD3 bispecific antibodies. Additional research and clinical trials are warranted to validate the efficacy of this combined therapeutic approach across various tumor types.

## 4 Conclusion

The initial results presented here offered preclinical support for the potential effectiveness of ICAM‐1–Dxd in combination with B7‐H3‐CD3 as a treatment strategy for TNBC, encouraging further clinical translational studies in ICAM‐1‐positive TNBC patients. This research also underscores the necessity for additional large‐scale studies to thoroughly evaluate the safety and efficacy of combining ICAM‐1–Dxd with B7‐H3‐CD3 in clinical settings. Moreover, this combination therapy strategy, including immune checkpoint bispecific antibodies, could be leveraged for the synergistic treatment of TNBC and other solid tumors that overexpress ICAM‐1. This approach has demonstrated substantial potential for application in future clinical trials, with a focus on TIM reprogramming.

## 5 Experimental Section

### Materials

ICAM‐1 mouse monoclonal antibodies (mAbs; sc‐8439) were purchased from Santa Cruz Biotechnology, Inc. (NC, USA). MC‐GFG‐Dxd (#1600418‐29‐8) and Obrindatamab (B7‐H3×CD3; #2069959‐72‐2) were acquired from MedChem Express (Shanghai, China). IgG rabbit mAbs (#ab133470) and Cyanine5.5 maleimide were obtained from Abcam Co. (Cambridge, England). CD276/B7‐H3 and CD3E antibodies were obtained from the Antibody System (Strasburg, France). The identities of TNBC cell lines (SUM159, MDA‐MB‐231, Hs578T, and BT‐549), a murine TNBC cell line (4T1), and the human mammary epithelial cell line MCF10A were verified through STR profiling. Rigorous testing confirmed that all the cells were devoid of mycoplasma contamination. In the laboratory, the cells were subcultured for 15 generations.

### Quantification of ICAM‐1 Expression

The protein abundance of ICAM‐1 in various cell types was determined through Western blot analysis. Furthermore, the presence of ICAM‐1 in human TNBC, 4T1, and MCF10A cell populations was measured via flow cytometric techniques. Briefly, a sample of 10^6^ cells was procured for each trial and subjected to three washes via the rotational washing technique. The cell samples were encapsulated in ice with 1% BSA and then exposed to Cy5.5‐conjugated antibodies at ambient temperature for 2 h. The samples were subsequently washed four times with PBS containing 1% FBS, followed by examination via flow cytometric techniques.

### Targeted Evaluation of Anti‐ICAM‐1 Antibodies for TNBC Cells

The specificity and binding affinity of the anti‐ICAM‐1 antibody for TNBC cells were evaluated in vitro via an ICAM‐1–Cy5.5‐conjugated antibody. The IgG–Cy5.5 antibody served as a control. The cells were first seeded into 8‐well plates, ensuring they were dispersed at a suitable volume; after 24 h, the existing growth medium was exchanged for one containing an ICAM‐1–Cy5.5‐conjugated antibody. Next, we maintained the cell environment at 37 °C for an additional 4 h. The cells were subsequently suspended in chilled PBS and stabilized with a 4% paraformaldehyde solution. To visualize the cell nuclei, we employed 4′,6‐diamidino‐2‐phenylindole hydrochloride in conjunction with an antifade mountant to enhance contrast and facilitate cellular identification. Images of the cellular structures were captured via a confocal laser scanning microscope.

### Western Blotting

The quantification of ICAM‐1 across various cell lines was performed via Western blot analysis. For protein extraction, 2 × 10^6^ cells were lysed in radioimmunoprecipitation assay buffer supplemented with protease inhibitors. Next, an estimated 35–40 µg of extracted protein from each sample was subjected to separation via SDS‒PAGE and subsequently transferred onto a nitrocellulose membrane. The membranes were treated with a 5% skim milk solution in TBS‐T to prevent nonspecific binding at ambient temperature for 1 h before being submerged in primary antibody solutions overnight. The primary antibodies utilized were anti‐ICAM‐1 and anti‐GAPDH, each at dilutions ranging from 1:400 to 1:2000. An Image Quant LAS 4000 from GE Healthcare Life Sciences was used to scan the blots.

### Immunohistochemistry

Newly obtained tissue samples were preserved with 4% paraformaldehyde, embedded in paraffin, and heated at 64 °C for 2 h. The sections were then deparaffinized with xylene before being progressively rehydrated through a descending gradient of ethanol solutions. The slices were subsequently heated in a uniform mixture of antigen retrieval solution until they solidified (12 min) to unmask the antigens, after which they were allowed to return to ambient temperature. The activity of innate peroxidases was inhibited using a 3% hydrogen peroxide solution. Per the protocols supplied within the assay kit, the samples were incubated overnight at 4 °C with the following primary antibodies: Ki67 (HA721115, HUABIO, diluted 1:100), CD31 (GB120005‐100, Servicebio, diluted 1:500), ICAM‐1 (sc‐8439, Santa Cruz, diluted 1:800), and TUNEL (G1504, Servicebio). Diaminobenzidine (DAB) was employed as the chromogenic substrate for the color reaction. The proportion of cells that exhibited positive staining was measured using ImageJ software for quantification.

### Enzyme‐Linked Immunosorbent Assay (ELISA)

Blood samples were taken from mice with tumors to measure IL‐10, IL‐12, IL‐6, TNF‐α, and IFN‐γ expression. The serum levels of these cytokines were quantified via ELISA kits (Elabscience, Wuhan, China) following per the manufacturer's directions.

### Cell Imaging Studies

Six‐well plates were utilized to culture the cells at a suitable density. The following day, the old medium was replaced with medium containing the ICAM‐1–Cy5.5 antibody, after which the cells were incubated for an additional 4 h at 37 °C. The cells were then resuspended in chilled PBS before being evaluated with an Image Stream X Mark II imaging flow cytometer (Seattle, Washington, USA).

### Assessment of ICAM‐1 Expression in Patient Samples

A total of 80 breast cancer samples were collected from individuals at Anhui Province Cancer Hospital, with each patient providing informed consent for inclusion in the research, per the tenets of the Helsinki Declaration. The study received ethical clearance from the Anhui Province Cancer Hospital's Ethics Committee (Approval no.: 2 022 237). IHC evaluations were conducted on a cohort of 40 human TNBC samples classified into three stages (stage I, 10 instances; stage III, 10 instances; stage IV, 10 instances) and an equal number of normal breast tissue samples to assess ICAM‐1 levels. Immunohistochemical staining quantification was performed via the H score, as previously described.^[^ ^28^ ^]^

### Construction of ADCs

The ICAM‐1–Dxd drug conjugate was prepared as previously described.^[^ ^29^ ^]^ The sodium bicine buffer (200 µL at 1 m, pH 8.0) and the sodium diethylenetriaminepentaacetic acid solution (20 µL at 100 mm, pH 7) were both supplemented with either ICAM‐1 antibody or IgG, each at a concentration and volume of 0.2 mg mL^−1^ and 500 µL, respectively. Following the initial steps, a reduction reaction was carried out using four equivalents of tris(carboxyethyl)phosphine (TCEP) for a duration of 2 h at 37 °C. After the completion of the reduction, the resulting solution was then combined with four equivalents of either maleimidocaproyl‐GGFG‐Dxd (MC‐GGFG‐Dxd) or Cyanine5.5 maleimide (MC‐Cy5.5) to proceed with the subsequent conjugation process. Following a 2 h period of agitation, the gel was separated using a filter (Sephadex G25, 1.0 g) and subsequently washed with PBS. Reaction surveillance was conducted using an apparatus comprising a high‐efficiency liquid chromatography (LC) capillary system (Ultimate 3000 from Thermo Fisher Scientific) and an accompanying electrospray ionization quadrupole time‐of‐flight mass spectrometer (MS) fitted with an ACQUITY UPLC Protein BEH C4 column (pore size 300 Å, particle size 1.7 µm, dimensions 2.1 mm by 50 mm). The method for eluting compounds was established as follows: solvent A consisted of water with 0.1% formic acid, whereas solvent B consisted of acetonitrile with a similar concentration of formic acid. The linear gradient profile began at 5% B, increased to 100% B over 10 min, held constant at 100% B for 5 min, then decreased back to 5% B over 2 min, and finally maintained at 5% B for an additional 2 min. The liquid was delivered at a constant speed of 0.3 mL per minute. The average DAR values were determined through LC‒MS analysis.

### Cytotoxicity Studies

Cells from triple‐negative breast cancer were propagated in slides with 96 chambers, each well containing 5000 cells. The medium was replaced with medium supplemented with nanoparticle Nab PTX, IgG–Dxd, or ICAM‐1–Dxd at various concentrations. After 72 h, the toxic effects on the cells were assessed via a CCK‐8 test. In brief, the medium containing the pharmaceuticals was discarded, and the cells were carefully rinsed with chilled PBS. The samples were subsequently incubated in a CCK‐8 solution maintained at 37 °C for 4 h. The degree to which cell growth was suppressed was determined by comparing the optical density of agent‐exposed cells to that of untreated control cells. In addition, extracellular ATP concentrations were quantified via a Beyotime ATP detection kit (S0026).

### Establishment of TNBC Organoid Cultures

TNBC organoid cultures were established as follows: neoplastic specimens were sectioned into small fragments and subjected to enzymatic disintegration using a specialized solution for digesting tumor tissue, which was maintained for 30 min at 37 °C within a shaking incubator with temperature control. The resulting single‐cell suspension was procured by straining through a 100‐µm filter. Individual cells encapsulated within Matrigel were subsequently plated in 24‐well plates with a nutritive medium conducive to growth.

Cytotoxicity studies in TNBC organoids were performed per a well‐established protocol.^[^ ^30^ ^]^ The organoids were treated with PBS, ICAM‐1, IgG–Dxd, or ICAM‐1–Dxd for three days, after which cell survival was assessed via the Cell Titer‐Glo 3D cell viability assay.

### In Vivo Targeting Functionality Assessment

Experiments to evaluate mouse tumor formation adhered to protocols sanctioned by the University of Science and Technology of China's Institutional Animal Care and Use Committee. ≈2 × 10^6^ TNBC cells (4T1) were inoculated into the subdermal region of the right rear flank of female BALB/c mice aged six weeks. By the 14th day, these cells had proliferated to create a tumor measuring 150 mm.^[^ ^3^ ^]^ The mice were subsequently divided into two equal cohorts of five and intravenously administered IgG–Cy5.5 or ICAM‐1–Cy5.5 (5 mg kg^−1^) through the caudal vein. At the 6, 12, and 24 h time points, in vivo, near‐infrared (NIR) fluorescence imaging was conducted via the IVIS Lumina II system (Waltham, MA, USA). Quantitative analysis was performed on the fluorescent signal detected across various organs, such as the heart, liver, spleen, lungs, kidneys, and tumor tissues.

### Pharmacokinetic Evaluation

BALB/c mice were randomly and evenly divided into seven groups (*n* = 3) for PK (pharmacokinetics, PK) analysis. Mice were injected with 5 mg kg^−1^ ICAM‐1‐Dxd via the tail once a week for a total of three doses. For day1‐7 and day15‐21, blood samples were collected at 0 h, 5 min, 1, 8, 24, 72, and 168 h. Plasma samples were analyzed using a validated ELISA method to quantitatively determine the total antibody and conjugated Dxd (anti‐Dxd antibody) (MedChemExpress, Shanghai, China). The data collected were subjected to analysis using the noncompartmental model within Winnonlin software to determine the key PK parameters.

### Bioinformatics Analysis of B7‐H3: Data Collection

The expression matrix, clinical information, and survival data of TNBC patients were extracted from the TCGA‐BRCA (https://portal.gdc.cancer.gov/) dataset. GSE76250 was used as the validation dataset.

### Bioinformatics Analysis of B7‐H3: Analysis of B7‐H3 Gene Expression

To investigate the expression levels of B7‐H3 and other related genes in TNBC and normal samples, we used the Wilcoxon rank‐sum test to compare the differences between the two groups (TCGA‐TNBC vs control). *p* < 0.05 was considered to indicate *significance*.

### Bioinformatics Analysis of B7‐H3: Gene Set Enrichment Analysis (GSEA)

We used data from the TCGA‐TNBC cohort to calculate the correlation between biomarkers of targeted genes via the R package “psych” and the Spearman method. The genes were then ranked according to their correlation coefficients from highest to lowest. We subsequently used the R package “clusterProfiler.” The reference gene set was c2.cp.kegg.v2023.1.Hs.symbols.gmt from the MSigDB database (https://www.gsea‐msigdb.org/gsea/msigdb).

### Bioinformatics Analysis of B7‐H3: Differential Expression Analysis

The DESeq2 package (v 1.38.0) was used to identify differentially expressed genes (DEGs) between the breast cancer and normal groups in the training set. DEGs were selected on the basis of the adjusted criteria. *p* < 0.05 and |log2fold change (FC)| > 1. Visualization of the DEGs was performed via ggplot2 (v. 3.4.4) for the volcano plot.

### Bioinformatics Analysis of B7‐H3: PPIs of Candidate Genes

The STRING database (https://string‐db.org) was used to predict relative PPIs, including direct and indirect physical interactions. Cytoscape 3.7.1 (https://www.cytoscape.org/) was used to construct a network of potential key targets and to perform a systematic analysis of the network parameters.

### Bioinformatics Analysis of B7‐H3: Drug Prediction

To explore potential treatments for TNBC, it was searched for potential drugs targeting the biomarker B7‐H3 in the drug–gene interaction (DGIdb) database (https://www.dgidb.org/org/), filtering drugs with a score >1.

### Bioinformatics Analysis of B7‐H3: Potential Therapeutic Drugs

To explore potential therapeutic drugs for TNBC, the DGIdb was used to search for targeted biomarkers. Potential drugs targeting B7‐H3 and related genes were screened (for drugs with scores >1).

B7‐H3‐related compounds were also explored, screening compounds with an interaction count of >1. Individuals with higher interaction frequencies were selected as research subjects. The mRNA interaction network was visualized via Cytoscape software. The optimal binding position of a compound to its target molecule was determined. Drugs with high B7‐H3 scores in the CTD database were selected for the molecular docking analysis.

### Antitumor Effects in 4T1 Tumor‐Bearing Models

TNBC cells from the 4T1 line were implanted into female BALB/c mice aged 6–8 weeks to evaluate in vivo antitumor activity. Once the tumors reached sizes ranging from 100 to 150 mm,^[^ ^3^ ^]^ the mice were randomly assigned to different cohorts, each consisting of 5 mice. The mice were subsequently injected with standardized amounts of PBS, B7‐H3‐CD3, ICAM‐1–Dxd, or a combination of ICAM‐1–Dxd and B7‐H3‐CD3. The tumor sizes were measured thrice weekly using an electronic caliper, and the volumes were calculated via the formula “tumor volume = length × width^2^/2.” Prior to the collection of blood through cardiac puncture, the mice with tumors were sedated with carbon dioxide. The blood sample was allowed to settle for 30 min before it was centrifuged for 15 min at 2000 × g to extract the serum. We subsequently measured the levels of AST, ALT, creatinine, and BUN using ELISA kits supplied by Huabangbio Co. (Shanghai, China). In addition, staining procedures, including H&E, TUNEL, ICAM‐1, CRT, and HMGB1, were conducted on tumor tissue slices. Various immune cell types within lung metastatic tumor sections were subsequently identified through immunofluorescence staining on the basis of established markers of immune cell subpopulations.

### TNBC PDX Studies

Peripheral blood mononuclear cells (PBMCs) were isolated from samples from healthy individuals via density gradient centrifugation. These cells were then cultured in RPMI 1640 medium supplemented with an antimicrobial antifungal compound and pyruvate at a concentration of 1 mm. PBMCs were incubated with TCR or IL‐2 for 3 days.

The procedures were approved by the Ethics Committee of Anhui Provincial Cancer Hospital. Freshly resected tumors from TNBC patients were collected during surgery. The tumors were placed in Dulbecco's Modified Eagle Medium supplemented with antibiotics at 0 °C. The tumor tissues were rinsed three times and cut into 3 × 3 × 3 mm pieces. Tumor cell suspensions mixed with Matrigel were inoculated subcutaneously into the right flank of 7‐week‐old female NOD/ShiLtJGPt*Prkdc^em26^* I*l2rg^em26^*/Gpt (NSG) mice. Upon tumor growth to a volume between 100 and 150 mm,^[^ ^3^ ^]^ the mice were randomly grouped (*n* = 6) and injected with 5 million PBMCs via caudal vein injection. Then, the mice were injected with or without established concentrations of PBS, B7‐H3‐CD3, ICAM‐1–Dxd, or ICAM‐1–Dxd plus B7‐H3‐CD3. The tumor volume was determined as described above. Meticulous tracking of the mouse body mass continued through to 21 days. On this day, the NSG mice were humanely euthanized, and their tumors were collected. The collected samples were subjected to H&E, Ki67, and CD31 staining and TUNEL immunofluorescence staining for CD4+ and CD8+ T cells. In addition, the concentrations of IL‐6, TNF‐α, and IFN‐γ in the serum of the NSG mice in each experimental group were measured via ELISA.

### Statistical Analysis

The statistical analyses were shown as averages with corresponding standard deviations (SD). Statistical evaluations were conducted using GraphPad Prism version 9, employing a two‐tailed Student's *t*‐*test* or an analysis of variance. Survival data were analyzed via the log‐rank test method. Thresholds for statistical significance are denoted by *p < 0.05, **p < 0.01, and ***p < 0.001.

## Conflict of Interest

The authors declare no conflict of interest.

## Supporting information



Supporting Information

## Data Availability

The data that support the findings of this study are available in the supplementary material of this article.
