# Supplementary material for: Reprogramming the Tumor Immune Microenvironment with ICAM‐1‐Targeted Antibody‒Drug Conjugates and B7‐H3‐CD3 Bispecific Antibodies
Source: Adv Sci (Weinh). 2025 Feb 25;12(16):2415577. doi: 10.1002/advs.202415577 (PMC12021053; doi:10.1002/advs.202415577)
Supplement: Supplementary file 1 — Supporting Information [file ADVS-12-2415577-s001.docx]

**Supplementary Information**

**Reprogramming the Tumor Immune Microenvironment with ICAM-1-Targeted Antibody‒Drug Conjugates and B7-H3-CD3 Bispecific Antibodies**


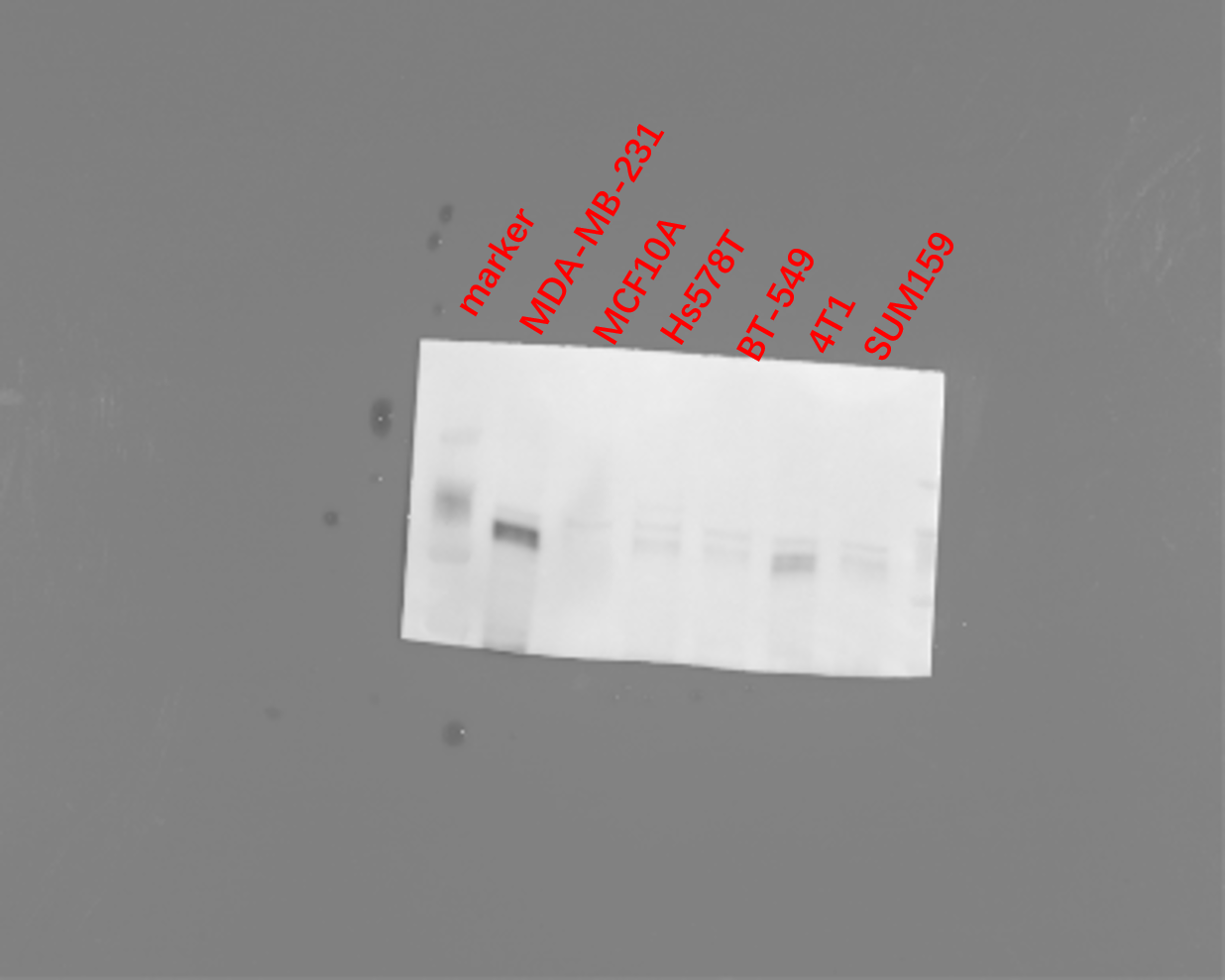

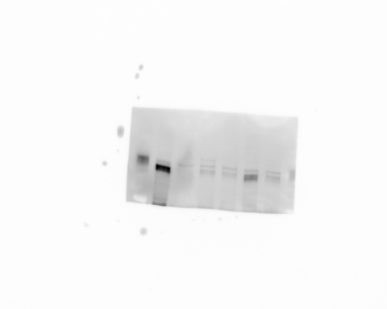


**Figure.S1.**  Western blotting original image of ICAM-1 in TNBC cell lines and MCF10A (as control).


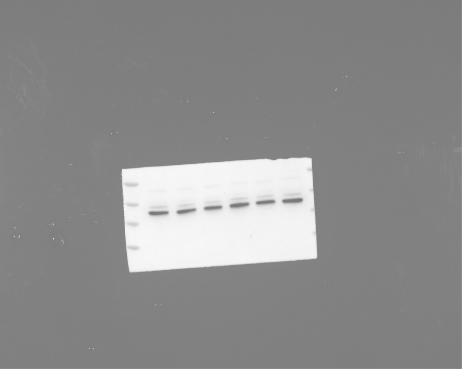

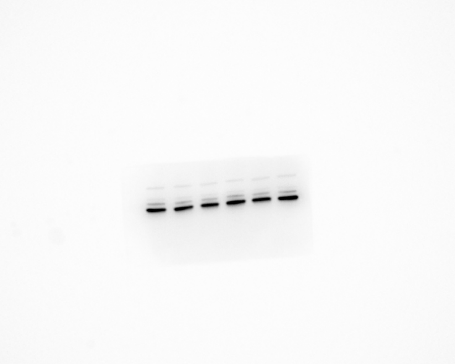


**Figure.S2.** Western blotting original image of GAPDH.


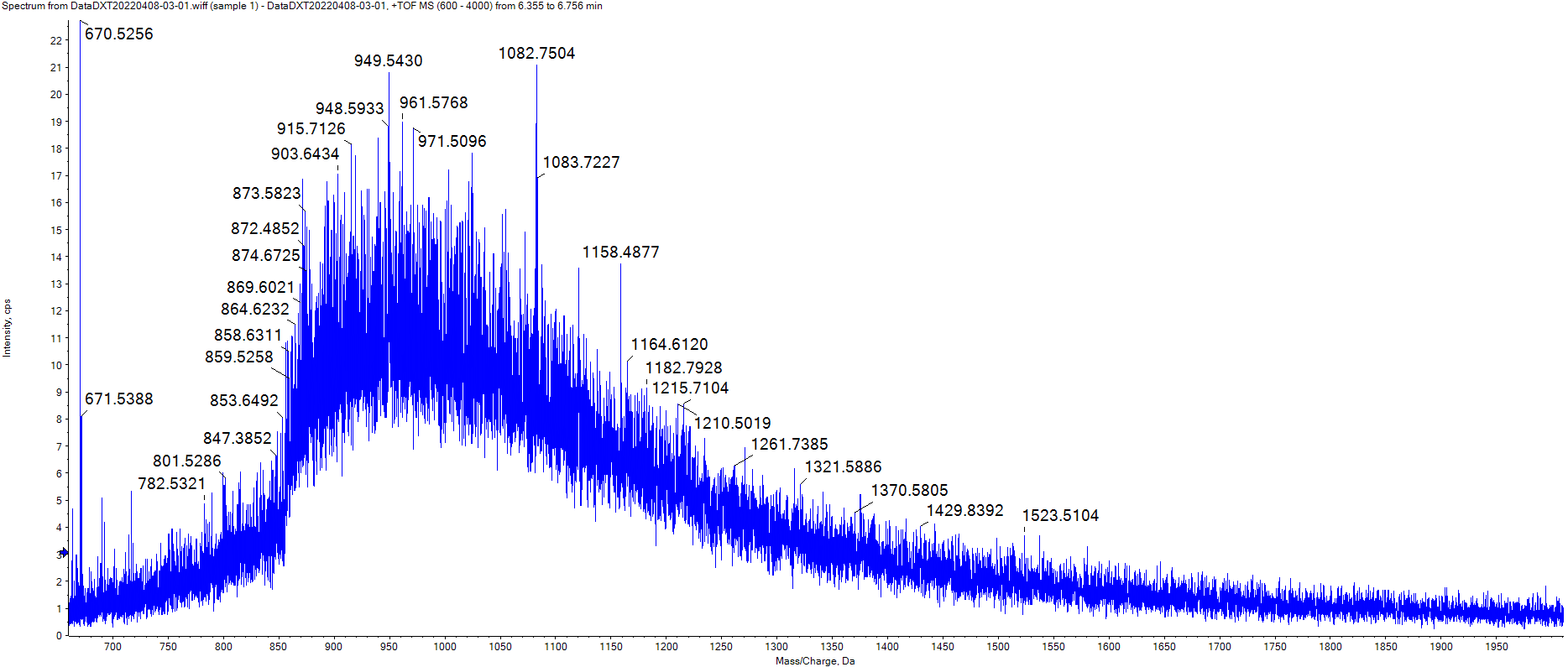


**Figure. S3.** Liquid chromatography-tandem mass spectrometry analysis of ICAM-1-Dxd.


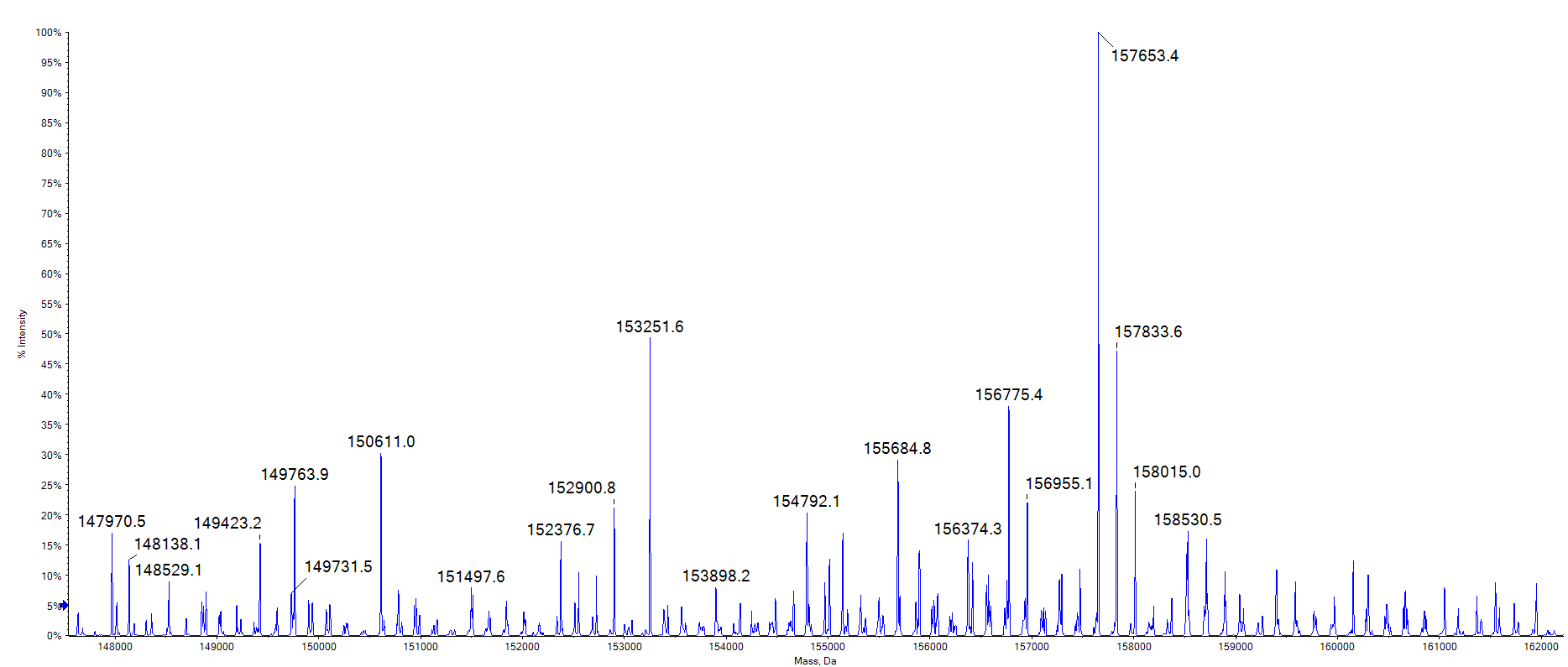


**Figure. S4.** Deconvolutional ESI-mass spectra analysis of ICAM-1-Dxd molecular weight.

| Average Mass | Sum Intensity | Relative Abundance% | Percentage% | | △Mass Da | Conjugation number of Dxd |
| --- | --- | --- | --- | --- | --- | --- |
| 147970.50 | 6336.24 | 22.55 | 3.38 | 5.84 | -29.50 | N/A |
| 148138.07 | 4606.40 | 16.39 | 2.46 |  | 138.07 | N/A |
| 148529.14 | 3278.44 | 11.67 | 1.75 | 1.75 | 529.14 | 1 |
| 149423.25 | 6788.32 | 24.16 | 3.62 | 9.99 | 1423.25 | 2 |
| 149731.48 | 3342.52 | 11.90 | 1.78 |  | 1731.48 | 2 |
| 149763.95 | 8572.33 | 30.51 | 4.58 |  | 1763.95 | 2 |
| 150611.01 | 12440.41 | 44.28 | 6.64 | 6.64 | 2611.01 | 3 |
| 151497.61 | 3275.49 | 11.66 | 1.75 | 1.75 | 3497.61 | 4 |
| 152376.70 | 4875.69 | 17.35 | 2.60 | 5.94 | 4376.70 | 5 |
| 152900.76 | 6247.00 | 22.23 | 3.34 |  | 4900.76 | 5 |
| 153251.56 | 13551.93 | 48.23 | 7.24 | 9.10 | 5251.56 | 6 |
| 153898.19 | 3486.05 | 12.41 | 1.86 |  | 5898.19 | 6 |
| 154792.11 | 9172.05 | 32.64 | 4.90 | 4.90 | 6792.11 | 7 |
| 155684.85 | 13021.49 | 46.34 | 6.95 | 6.95 | 7684.85 | 8 |
| 156374.29 | 7619.22 | 27.12 | 4.07 | 16.44 | 8374.29 | 9 |
| 156775.37 | 15022.68 | 53.47 | 8.02 |  | 8775.37 | 9 |
| 156955.12 | 8146.30 | 28.99 | 4.35 |  | 8955.12 | 9 |
| 157653.43 | 28097.79 | 100.00 | 15.00 | 23.63 | 9653.43 | 10 |
| 157833.56 | 16157.59 | 57.50 | 8.63 |  | 9833.56 | 10 |
| 158015.02 | 7744.55 | 27.56 | 4.14 | 7.07 | 10015.02 | 11 |
| 158530.49 | 5502.15 | 19.58 | 2.94 |  | 10530.49 | 11 |

| **Figure. S5.** Conjugation number of Dxd to ICAM-1 antibody(average DAR value=4.8). |
| --- |

| 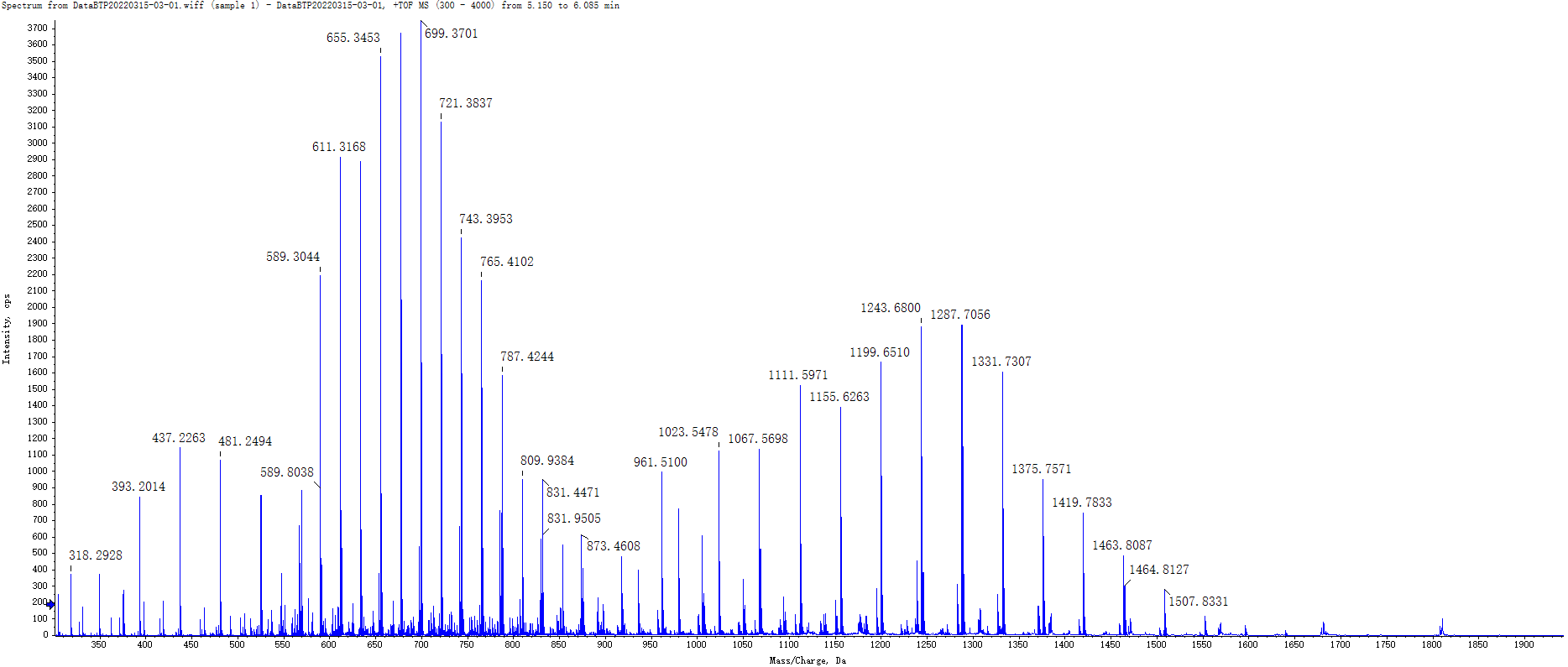 |
| --- |
|  |

**Figure. S6.** Liquid chromatography-tandem mass spectrometry analysis of ICAM-1-Cy5.5.


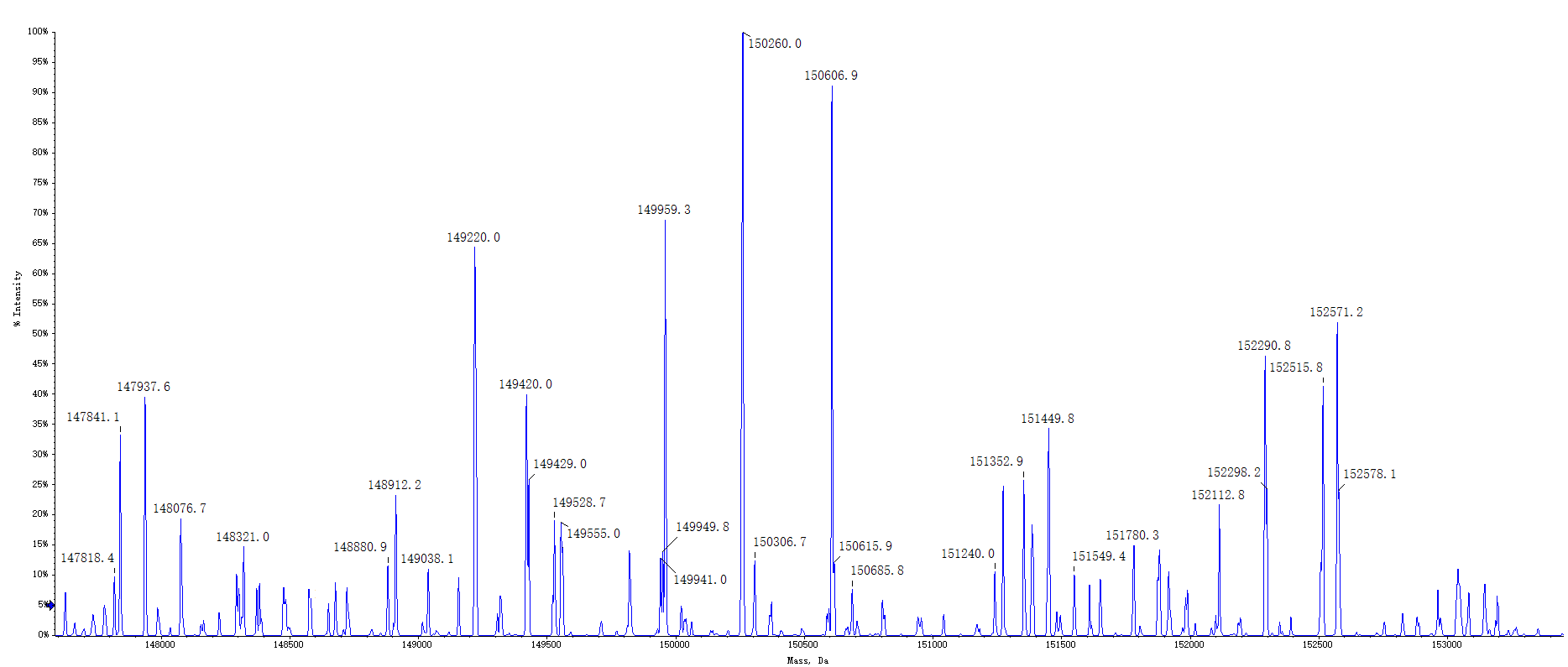


**Figure. S7.** Deconvolutional ESI-mass spectra analysis of ICAM-1-Cy5.5 molecular weight.

| Average Mass | Sum Intensity | Relative Abundance% | Percentage% | | △Mass Da | Conjugation number of Cy5.5 |
| --- | --- | --- | --- | --- | --- | --- |
| 147818.38 | 3591.58 | 7.07 | 0.93 | 11.99 | -181.60 | N/A |
| 147841.06 | 11952.96 | 23.51 | 3.08 |  | -158.90 | N/A |
| 147937.57 | 16187.25 | 31.84 | 4.18 |  | -62.40 | N/A |
| 148076.72 | 9390.54 | 18.47 | 2.42 |  | 76.70 | N/A |
| 148320.95 | 5349.93 | 10.52 | 1.38 |  | 321.00 | N/A |
| 148880.91 | 4780.70 | 9.40 | 1.23 | 20.93 | 880.90 | 1 |
| 148912.21 | 9363.40 | 18.42 | 2.42 |  | 912.20 | 1 |
| 149038.10 | 4688.35 | 9.22 | 1.21 |  | 1038.10 | 1 |
| 149220.01 | 38151.98 | 75.05 | 9.84 |  | 1220.00 | 1 |
| 149419.99 | 17284.63 | 34.00 | 4.46 |  | 1420.00 | 1 |
| 149429.03 | 6825.34 | 13.43 | 1.76 |  | 1429.00 | 1 |
| 149528.75 | 8329.69 | 16.39 | 2.15 | 27.15 | 1528.70 | 2 |
| 149554.96 | 7197.54 | 14.16 | 1.86 |  | 1555.00 | 2 |
| 149941.00 | 3963.72 | 7.80 | 1.02 |  | 1941.00 | 2 |
| 149949.82 | 4020.92 | 7.91 | 1.04 |  | 1949.80 | 2 |
| 149959.31 | 25586.30 | 50.33 | 6.60 |  | 1959.30 | 2 |
| 150259.96 | 50832.89 | 100.00 | 13.12 |  | 2260.00 | 2 |
| 150306.74 | 5299.74 | 10.43 | 1.37 |  | 2306.70 | 2 |
| 150606.94 | 29937.39 | 58.89 | 7.73 | 17.89 | 2606.90 | 3 |
| 150615.86 | 3771.84 | 7.42 | 0.97 |  | 2615.90 | 3 |
| 150685.84 | 3283.76 | 6.46 | 0.85 |  | 2685.80 | 3 |
| 151240.01 | 4446.98 | 8.75 | 1.15 |  | 3240.00 | 3 |
| 151352.93 | 10617.43 | 20.89 | 2.74 |  | 3352.90 | 3 |
| 151449.80 | 17268.96 | 33.97 | 4.46 |  | 3449.80 | 3 |
| 151549.37 | 4249.25 | 8.36 | 1.10 | 15.45 | 3549.40 | 4 |
| 151780.28 | 7155.65 | 14.08 | 1.85 |  | 3780.30 | 4 |
| 152112.75 | 6894.11 | 13.56 | 1.78 |  | 4112.80 | 4 |
| 152290.81 | 19572.89 | 38.50 | 5.05 |  | 4290.80 | 4 |
| 152298.18 | 5171.00 | 10.17 | 1.33 |  | 4298.20 | 4 |
| 152515.85 | 16836.50 | 33.12 | 4.34 |  | 4515.80 | 4 |
| 152571.19 | 19073.05 | 37.52 | 4.92 | 6.59 | 4571.20 | 5 |
| 152578.12 | 6461.61 | 12.71 | 1.67 |  | 4578.10 | 5 |

**Figure. S8.** Conjugation number of Cy5.5 to ICAM-1 antibody.


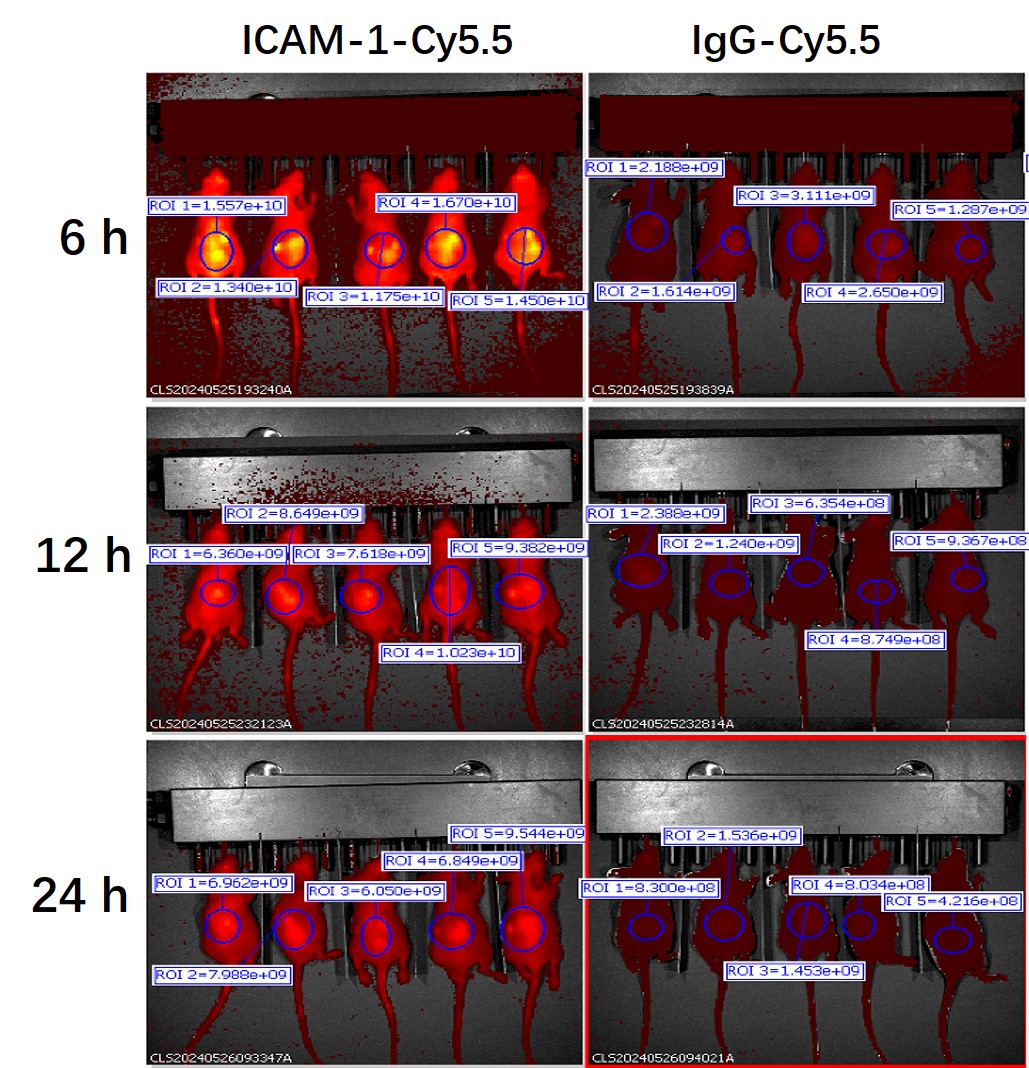


**Figure.S9.** Quantified of fluorescence intensity of mouse models at 6, 12, or 24h after intravenous injection IgG-Cy5.5 or ICAM-1-Cy5.5 (*n* = 5)


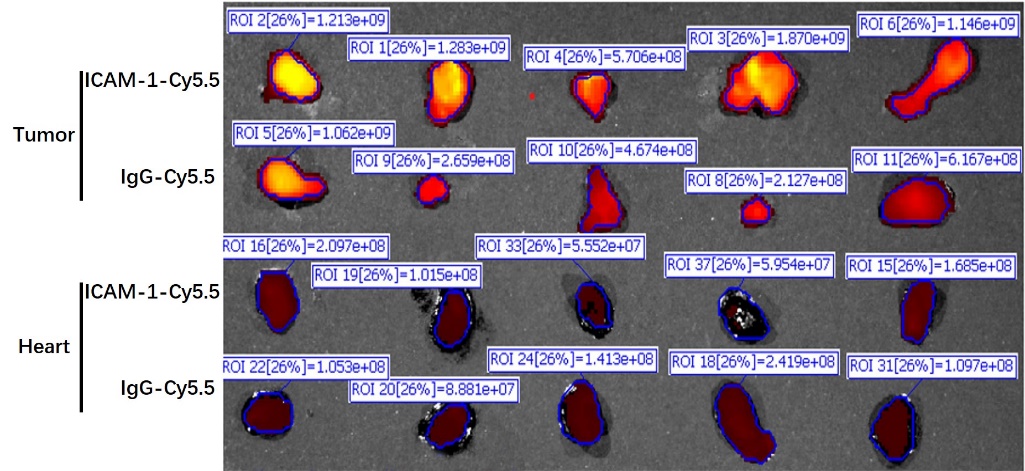


**Figure.S10.** Ex vivo quantified of fluorescence intensity of 4T1 tumors and hearts (*n* = 5).


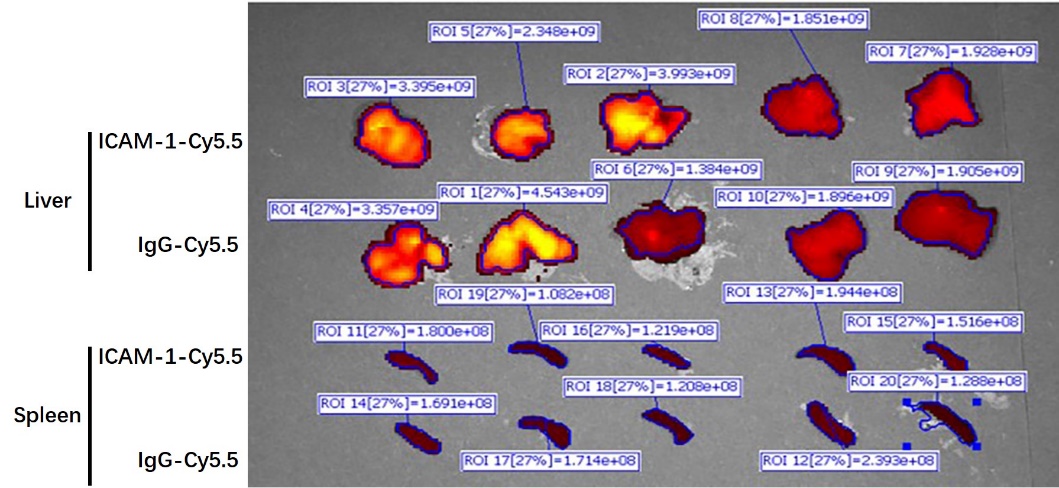


**Figure.S11.** Ex vivo quantified of fluorescence intensity of live and spleen (*n* = 5).


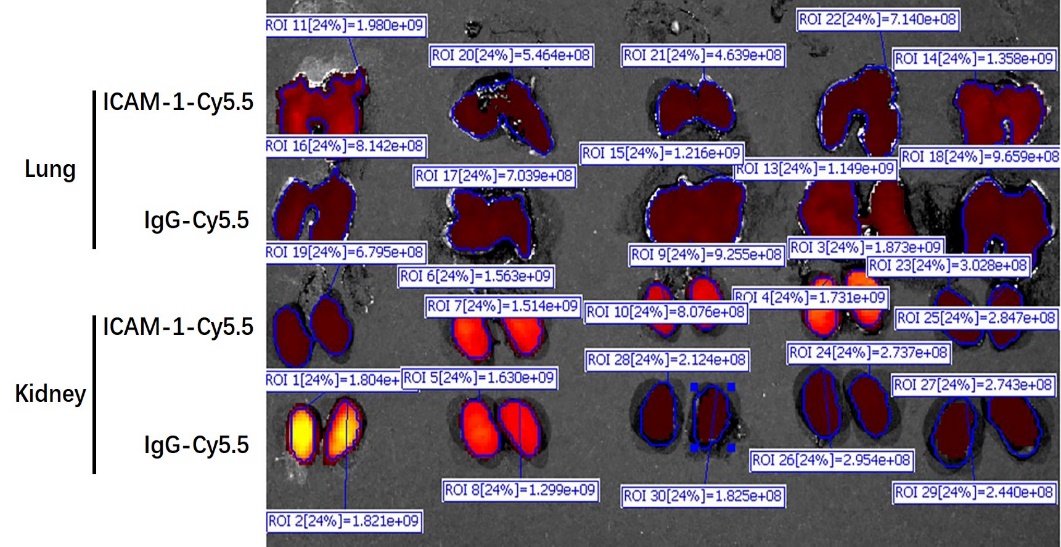


**Figure.S12.** Ex vivo quantified of fluorescence intensity of lung and kidney (*n* = 5).


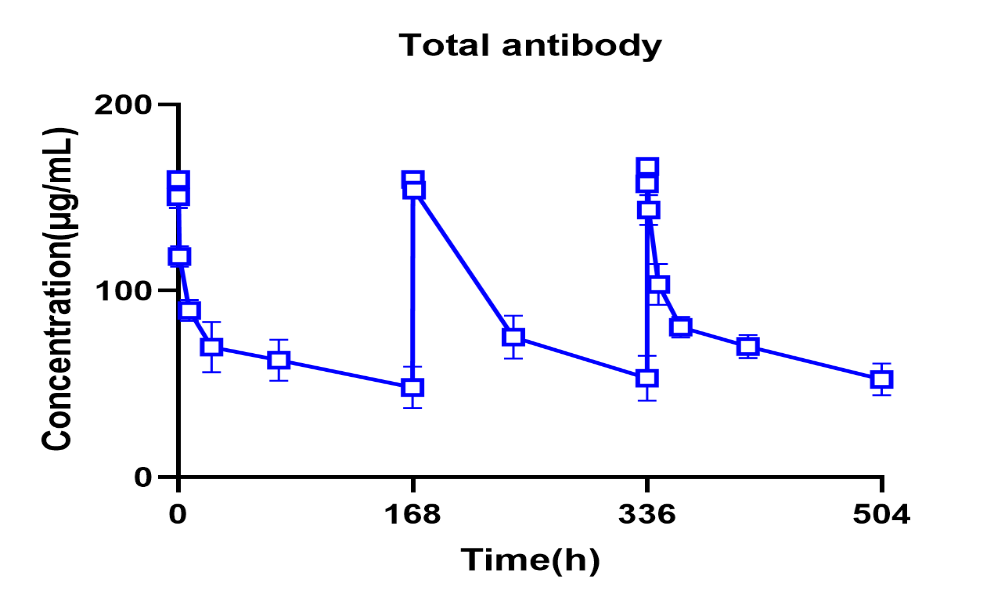


**Figure. S13. Repeated doses pharmacokinetic study of ICAM-1-Dxd (Total antibody).** ICAM-1-Dxd (5 mg/kg) was administered intravenously once a week for a total of three doses. Concentrations of total antibody in plasma were analyzed by ELISA. Data are expressed as Mean ± SD (*n* = 3).


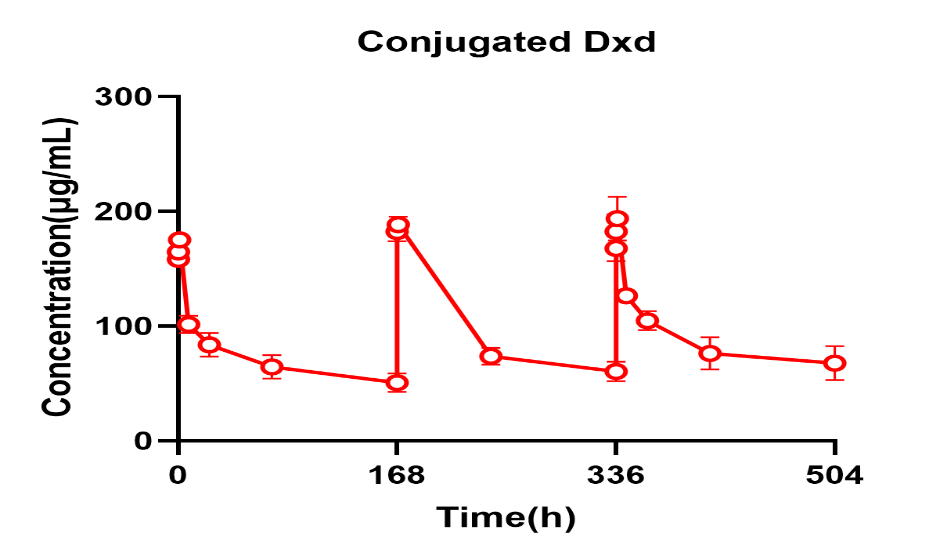


**Figure. S14. Repeated doses pharmacokinetic study of ICAM-1-Dxd (****Conjugated Dxd).** ICAM-1-Dxd (5 mg/kg) was administered intravenously once a week for a total of three doses. Concentrations of conjugated Dxd in plasma were analyzed by ELISA. Data are expressed as Mean ± SD (*n* = 3).


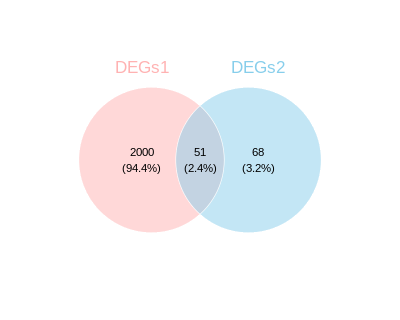


**Figure.S15.** Venn diagram of candidate genes.


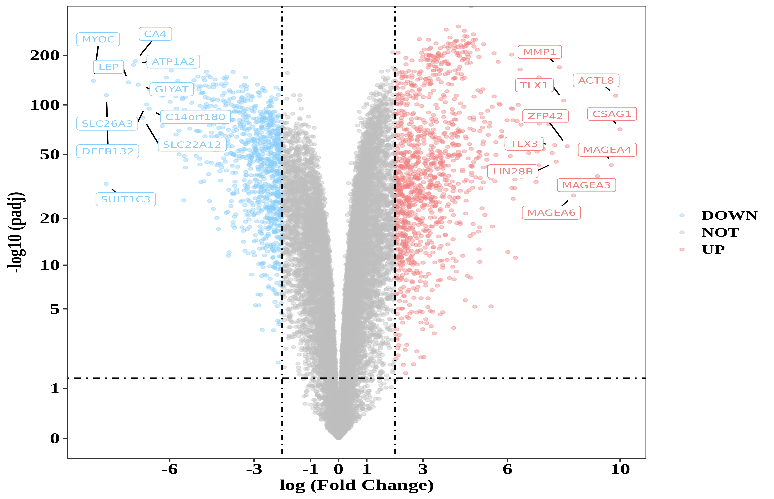


**Figure.S16.** Volcano plot of differential genes (TNBC vs Control).


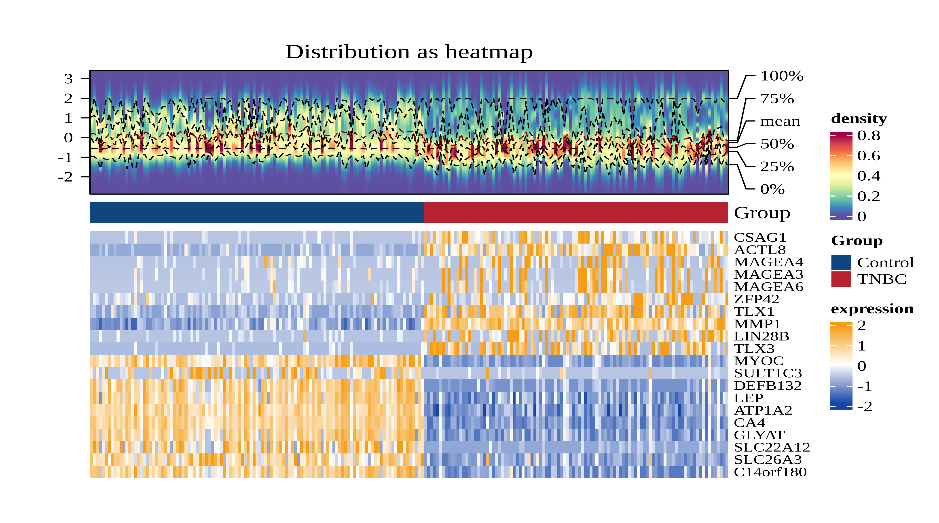


**Figure.S17.** Heat map of differential genes (TNBC vs Control).


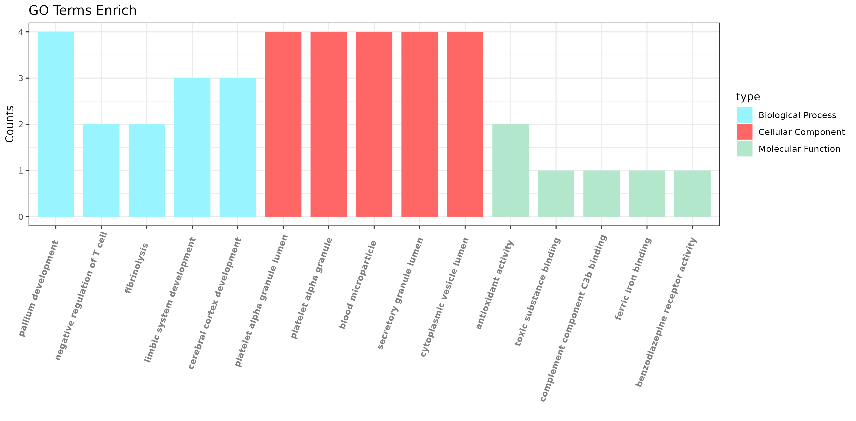


**Figure.S18.** GO enrichment analysis plot of candidate genes (The horizontal axis represents different functions, and the vertical axis represents the number of genes enriched).


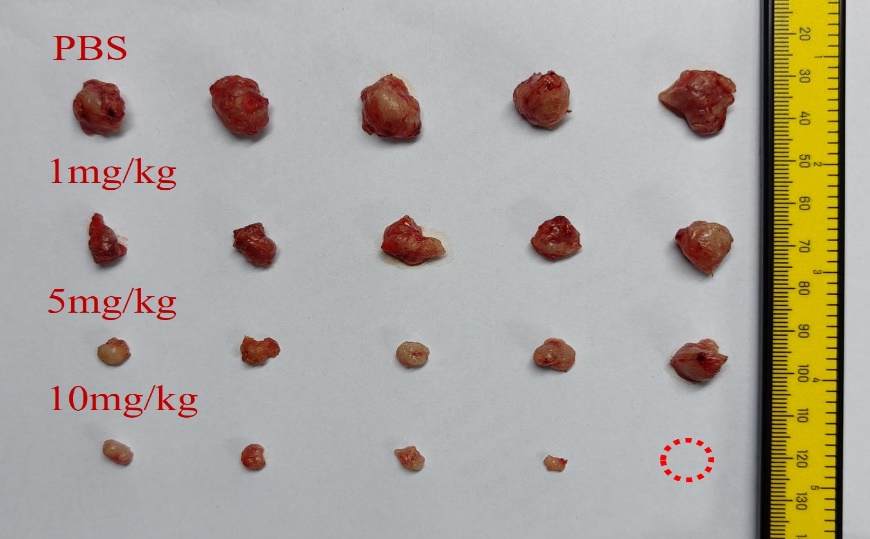


**Figure.S19.** Explore the optimal dosing based on ICAM-1-Dxd concentration gradient (1, 5, or 10 mg/kg). Ex vivo mouse tumor imaging (*n* = 5).


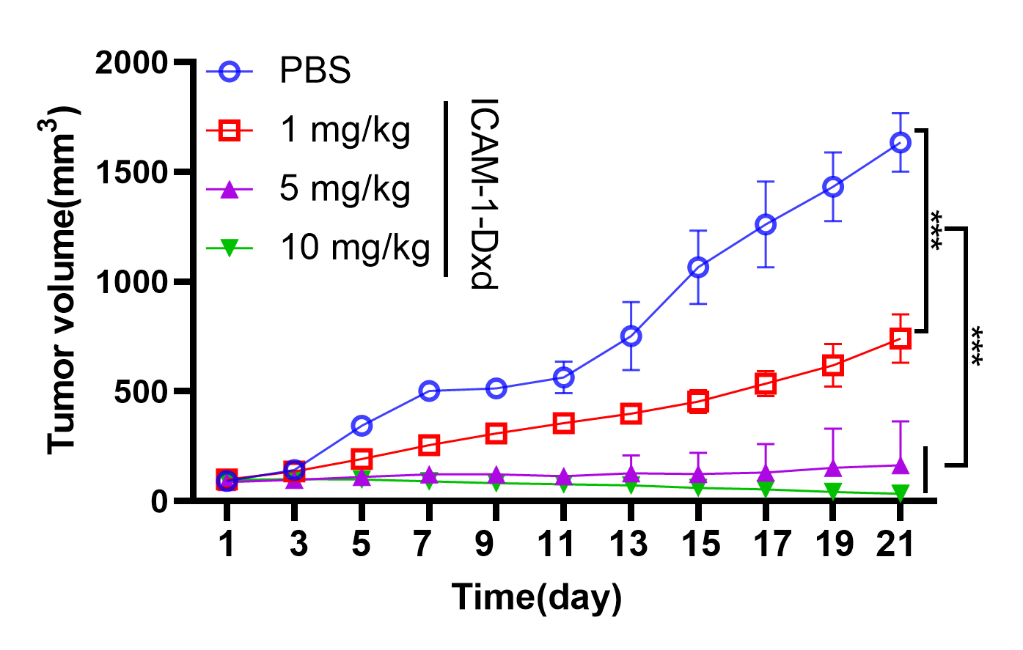


**Figure.S20.** Explore the optimal dosing based on ICAM-1-Dxd concentration gradient (1, 5, or 10 mg/kg). Changes in the volume of the tumors. ****p* <0.001.


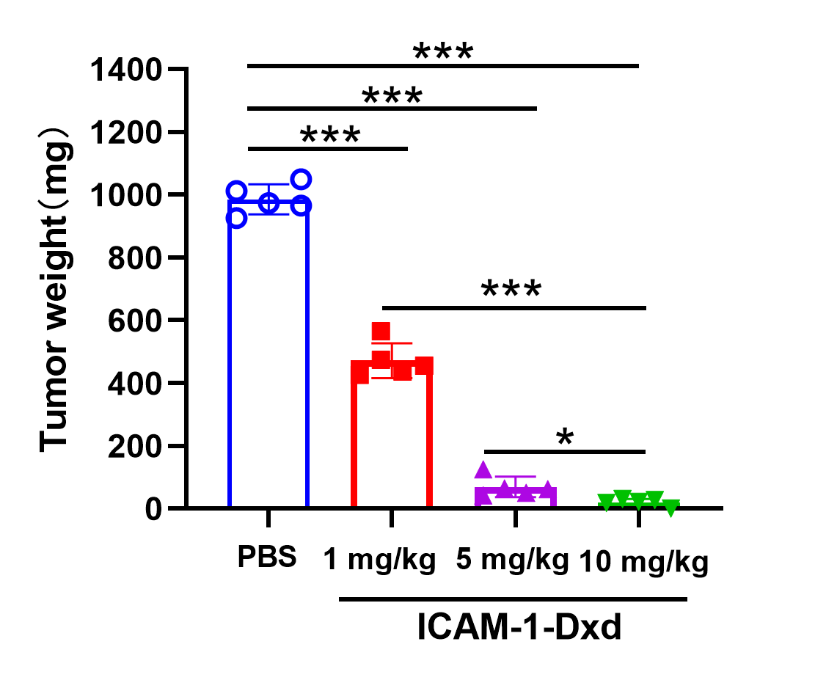


**Figure.S21.** Explore the optimal dosing based on ICAM-1-Dxd concentration gradient (1, 5, or 10 mg/kg). The tumor weights across different groups. **p* <0.05, ****p* <0.001.


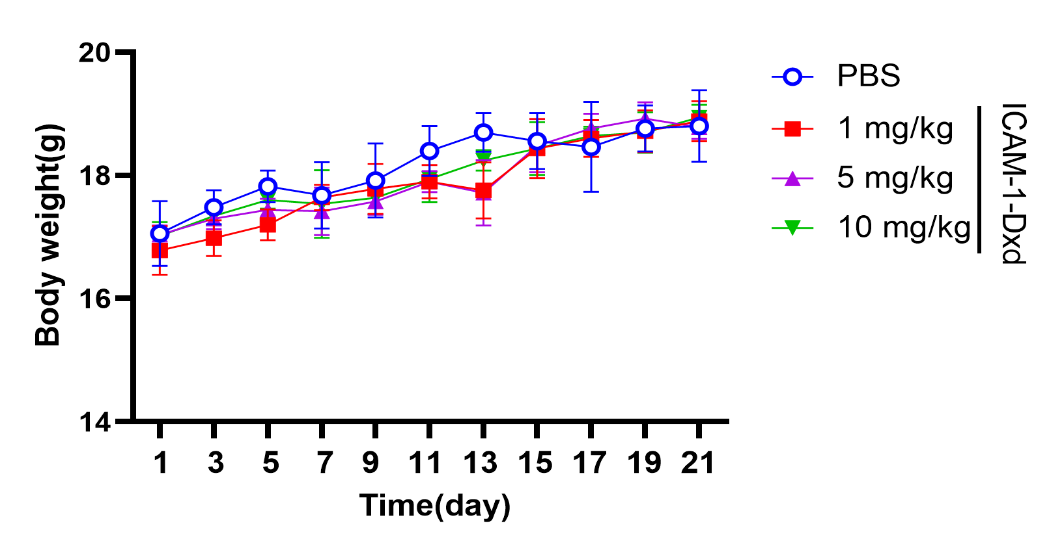


**Figure.S22.** Explore the optimal dosing based on ICAM-1-Dxd concentration gradient (1, 5, or 10 mg/kg). Curve of weight change in the mice.


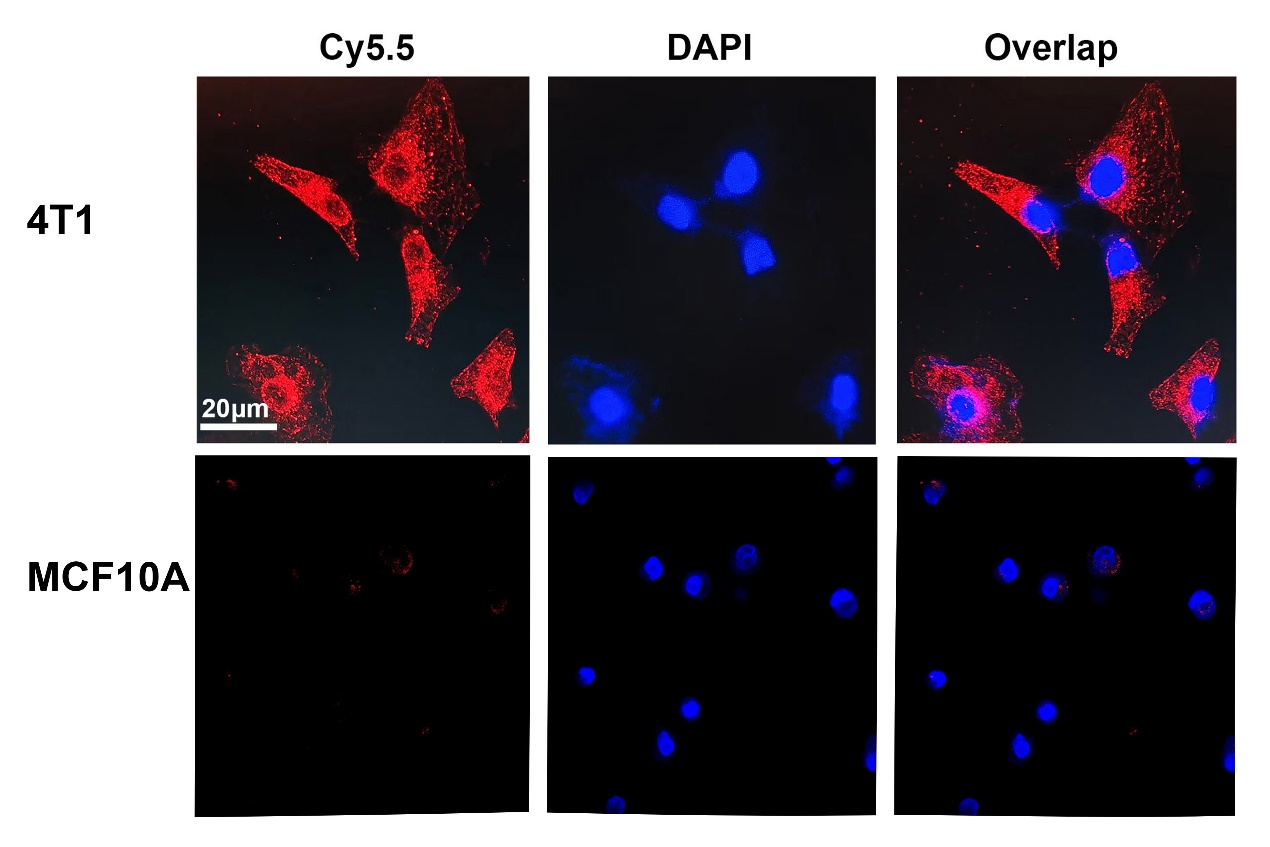


**Figure.S23.** Immunofluorescence staining of B7-H3 in 4T1 cells (MCF10A cells as a control).


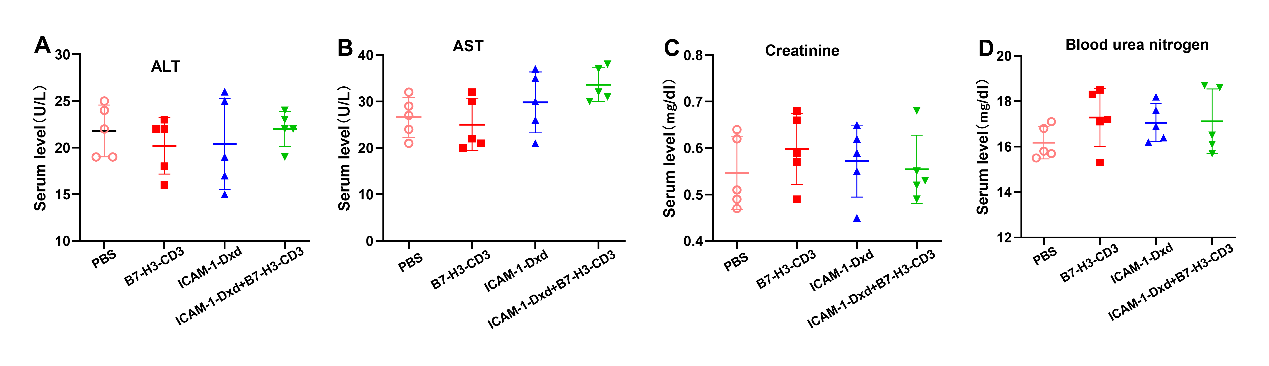


**Figure.S24.** Assessment of hepatotoxicity and nephrotoxicity via blood biochemistry. Quantitative analysis of ALT (A), AST (B), Creatinine (C), and blood urea nitrogen (D).


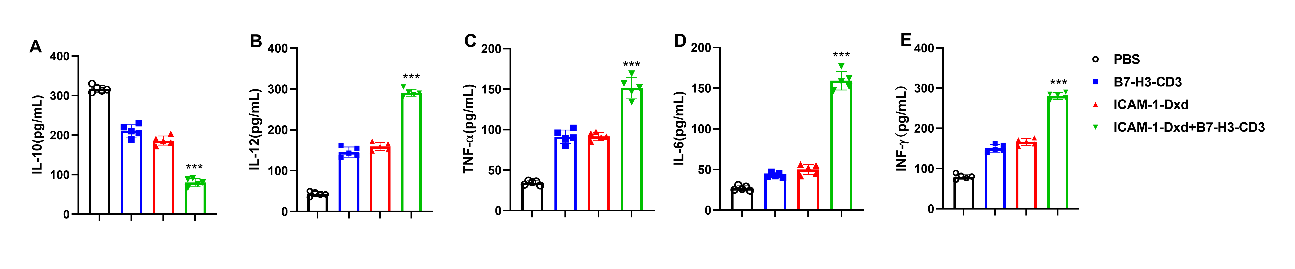


**Figure.S25.** Analysis of cytokines in 4T1 tumor-bearing mice. (A) Cytokine concentration of interleukin 10 (IL-10) in serum (*n* = 5). (B) Cytokine concentration of interleukin 12 (IL-12) in serum (*n* = 5). (C) Cytokine concentration of tumor necrosis factor-(TNF-α) in serum (*n* = 5). (D) Cytokine concentration of interleukin 6 (IL-6) in serum (n = 5). (E) Cytokine concentration of interferon-γ (INF-γ) in serum (*n* = 5). ***P < 0.001.


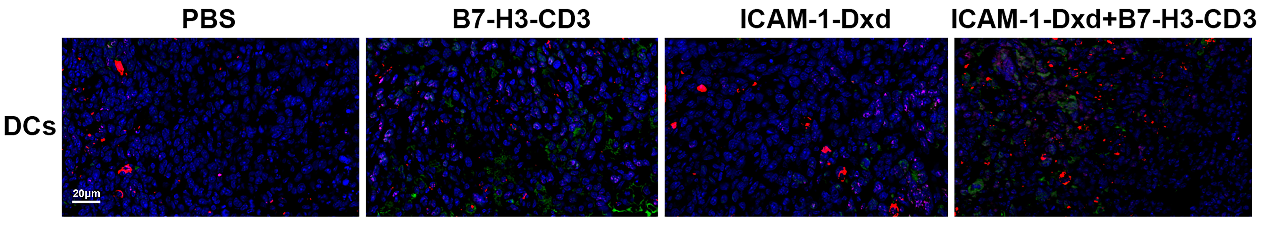


**Figure.S26.** Representative imaging of immunofluorescence staining of dendritic cells (DCs)in lymph nodes. DCs: CD11c+(red)/CD80+(green)/CD86+(pink), cell nucleus was stained with DAPI (blue).


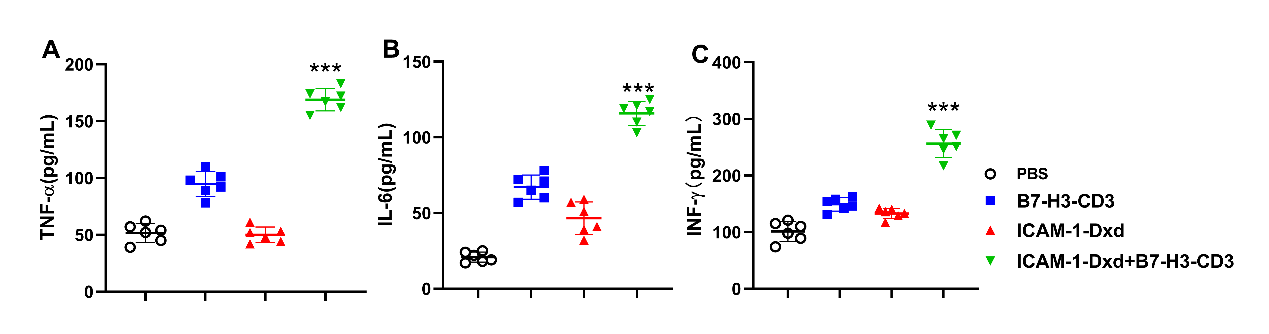


**Figure.S27.** Analysis of cytokines in PDX mice. (A) Cytokine concentration of tumor necrosis factor-α (TNF-α) in serum (*n* = 6). (B) Cytokine concentration of interleukin 6 (IL-6) in serum (*n* = 6). (C) Cytokine concentration of interferon-γ (INF-γ) in serum (*n* = 6). ****P* < 0.001.

**Table S1.** Pharmacokinetic parameters of total antibodies and conjugated Dxd in mouse serum after iv administration of 5 mg/kg ICAM-1-Dxd, once a week for a total of three doses (day1-day7).

| Parameters Units | |  | Drugs |
| --- | --- | --- | --- |
|  |  | Total antibodies | Conjugated Dxd |
| T_1/2β_ | h | 148.49 ± 1.08 | 139.42 ± 3.27 |
| T _max_ | h | 0.09 ± 0.04 | 0.89 ± 0.36 |
| C _max_ | µg/mL | 159.13 ± 41.17 | 178.13 ± 15.47 |
| AUC_0-168h_ | h*µg/mL | 10741.7 ± 449.85 | 12577.62 ± 372.13 |
| MRT_0-168h_ | h | 69.11 ± 2.36 | 66.58 ± 1.31 |
| CL | mL/h/kg | 0.63 ± 0.01 | 0.41 ± 0.03 |
| Vd | mL/kg | 81.54 ± 3.76 | 77.82 ± 1.54 |

Data are expressed as Mean ± SD.

**Table S2.** Pharmacokinetic parameters of total antibodies and conjugated Dxd in mouse serum after iv administration of 5 mg/kg ICAM-1-Dxd, once a week for a total of three doses (day15-day21).

| Parameters Units | |  | Drugs |
| --- | --- | --- | --- |
|  |  | Total antibodies | Conjugated Dxd |
| T_1/2β_ | h | 161.52 ± 0.89 | 168.48 ± 2.51 |
| T _max_ | h | 0.12 ± 0.03 | 0.65 ± 0.41 |
| C _max_ | µg/mL | 170.23 ± 44.89 | 208.26 ± 22.39 |
| AUC_0-168h_ | h*µg/mL | 11718.44 ± 466.65 | 13895.24 ± 402.92 |
| MRT_0-168h_ | h | 68.23 ± 3.47 | 70.21 ± 5.83 |
| CL | mL/h/kg | 0.52 ± 0.03 | 0.33 ± 0.02 |
| Vd | mL/kg | 61.31± 2.46 | 51.29 ± 3.17 |

Data are expressed as Mean ± SD.
